# Evolutionary Divergence, Predicted Interaction Interface, and Regulatory Specialization of MTB as a Non-Catalytic Scaffold in the Plant m6A Writer Complex

**DOI:** 10.3390/cimb48070722

**Published:** 2026-07-15

**Authors:** Hariharan Balasubramaniam, Susiharan Govindasamy Srinivasan, A. Santhana Krishna Kumar

**Affiliations:** 1 Biotechnology Center in Southern Taiwan, Academia Sinica, No. 100, Guiren 13th Road, Guiren District, Tainan 711010, Taiwan; hareharan.b@gmail.com; 2 Department of Chemistry, National Sun Yat-sen University, No. 70, Lien-hai Road, Gushan District, Kaohsiung 80424, Taiwan; susiharan.gs2006@gmail.com

**Keywords:** N6-methyladenosine, m6A writer complex, MTA70, MTB, land plant evolution, AlphaFold2, protein–protein docking, epitranscriptomic regulation, ABA signaling

## Abstract

N6-methyladenosine (m6A) is the most prevalent internal modification of eukaryotic mRNA and a central regulator of plant development and stress adaptation. The plant m6A writer complex requires two MT-A70 family proteins, the catalytic subunit MTA70 and its non-catalytic partner MTB, yet the evolutionary basis and structural logic underlying this functional division remain unresolved across land plant lineages. Here, we present an integrative computational analysis of MTA70 and MTB across 15 phylogenetically representative species spanning bryophytes, lycophytes, charophyte algae, monocots, and dicots. Phylogenomic reconstruction resolved three strongly supported clades, namely MTA70, MTB, and an intermediate MTA70-like group, demonstrating that catalytic-to-regulatory divergence predates the separation of major land plant lineages. MTA70 proteins exhibited strict conservation of gene structure, catalytic motifs, and domain architecture, reflecting selective constraint at functionally critical residues, whereas MTB showed extensive divergence in exon–intron organization and surface-exposed residues, consistent with relaxed structural constraints. AlphaFold2-based structural modeling and data-driven protein–protein docking predicted a stable MTA70–MTB heterodimer with a buried surface area of 1435 Å^2^ and a binding free energy of −8.1 kcal/mol, with Lys746 and Lys637 of MTB identified as primary interface hotspots by computational alanine scanning. Expression profiling across six species revealed preferential MTB accumulation in reproductive tissues, while promoter analysis identified statistically significant enrichment of jasmonate-responsive elements (TGACG-motif) in MTB promoters (Mann–Whitney U, *p* = 0.025) and a 3.4-fold higher abundance of ABA-responsive elements (ABRE) in MTB relative to MTA70, suggesting potential responsiveness to multiple phytohormone signals. Together, these findings establish an evolutionary and regulatory framework for MTB as a conserved scaffold coupling m6A deposition to developmental and environmental signaling in land plants.

## 1. Introduction

N6-methyladenosine (m6A) is the most abundant internal modification in eukaryotic mRNA and a central regulator of post-transcriptional gene expression, influencing pre-mRNA splicing, nuclear export, translation efficiency, and transcript stability [1]. In plants, m6A controls key developmental processes including embryogenesis, shoot meristem identity, and flowering time, and mediates responses to abiotic and biotic stresses [2,3,4].

The m6A modification is deposited by a conserved multi-subunit writer complex whose catalytic core belongs to the MT-A70 protein family. In mammals, the catalytic subunit METTL3 pairs with the non-catalytic METTL14 to form a heterodimer in which METTL14 functions as a structural scaffold that enhances RNA substrate binding without contributing to catalysis [5,6]. In *Arabidopsis thaliana*, MTA70 serves as the METTL3 ortholog and catalytic component, while MTB functions as the METTL14 equivalent. Loss-of-function mutants of both subunits display severe developmental defects, confirming that MTB plays an indispensable non-catalytic role in maintaining writer complex activity [2,7]. The plant complex additionally includes FIP37, VIRILIZER, and the E3 ubiquitin ligase HAKAI [7].

Despite these insights from model species, the evolutionary history of the MT-A70 family across land plants remains poorly understood. It is unclear whether the functional division between catalytic MTA70 and regulatory MTB is an ancient conserved feature or a lineage-specific innovation. Furthermore, a third intermediate clade designated MTA70-like occupies a phylogenetically ambiguous position between canonical MTA70 and MTB, and its evolutionary origin, whether representing an ancestral intermediate, a lineage-specific duplication, or a functionally distinct paralog, remains unresolved [8]. Genes encoding critical catalytic functions typically exhibit conserved exon-intron structures, domain arrangements, and sequence motifs under strong purifying selection, whereas non-catalytic regulatory subunits often show greater sequence divergence and domain reconfiguration associated with neofunctionalization [9,10]. Whether these evolutionary principles apply to MTA70 and MTB across bryophytes, monocots, and dicots remains to be systematically examined.

The structural basis of the plant MTA70-MTB interaction remains uncharacterized, and differences in promoter architecture between MTA70 and MTB may reflect distinct regulatory programs controlling m6A deposition across developmental and stress contexts [11]. Co-expression analysis of MTB in Arabidopsis can further reveal biological processes beyond its established role in the writer complex.

Here, we present an integrative computational analysis of MTA70 and MTB proteins across 15 representative lineages, integrating phylogenomics, gene structure and domain characterization, conserved motif identification, AlphaFold2-based structural modeling, protein-protein docking, promoter *cis*-element profiling, and cross-species expression analysis. Species selection was restricted to 15 lineages, including newly incorporated lycophyte (*Selaginella moellendorffii*) and charophyte algae (*Klebsormidium nitens*) sequences to maximize alignment quality and phylogenetic resolution, as inclusion of additional divergent sequences introduced topological instability without improving clade definition [8]. All structural, docking, and interaction analyses presented in this study are computational predictions and are intended as hypothesis-generating observations to guide future experimental work. Specifically, the predicted MTA70–MTB heterodimer interface geometry, the identification of Lys746 and Lys637 as interface hotspots by computational alanine scanning, the binding free energy estimate of −8.1 kcal/mol, and the codon selection analysis results from FUBAR represent predictions that require biochemical validation. By contrast, the indispensability of both MTA70 and MTB for m6A deposition in plants [2,7] and the structural conservation of the MT-A70 fold between plant and mammalian m6A writer complex subunits are established by existing experimental literature. This study establishes an evolutionary and structural framework for understanding how MTB functions as a conserved regulatory scaffold in the plant m6A writer complex.

## 2. Materials and Methods

### 2.1. Identification and Sequence Retrieval of MT-A70 Family Genes

Coding sequences (CDS), protein sequences, and corresponding genomic DNA sequences of MT-A70, MTA70-like, and MTB genes were retrieved from publicly available databases, including the NCBI, Phytozome, and UniProt [12,13,14]. Initial identification was based on annotated gene names and protein descriptions, which was further refined using homology-based searches. To ensure comprehensive detection of homologs across diverse plant lineages, homology searches were conducted using BLASTP 2.17.0+ with *Arabidopsis thaliana* MTA and MTB proteins as queries, with an E-value threshold of 1 × 10^−5^. Only full-length sequences containing intact MT-A70 domains were retained for downstream analyses. Protein sequences were used for phylogenetic, motif, and structural analyses, whereas CDS, genomic sequences, and gene annotation files (GFF3 format) were used for gene structure and promoter analyses.

### 2.2. Phylogenetic Analysis

To investigate the evolutionary relationships and divergence patterns of MT-A70 family proteins, phylogenetic analysis was performed using protein sequences from 15 representative species. Protein sequences were aligned using the MUSCLE algorithm implemented in MEGA version 11 to ensure accurate alignment of conserved domains [15,16]. The resulting alignment was used to construct a Maximum Likelihood phylogenetic tree based on the Jones–Taylor–Thornton (JTT) substitution model, selected according to the lowest Bayesian Information Criterion (BIC) score. Full-length protein sequences were used to preserve domain architecture, and the final alignment spanned 1–1444 amino acid positions. Statistical support for tree topology was assessed using 1000 bootstrap replicates, and only values greater than or equal to 70% are shown. The resulting tree was exported in Newick format and visualized using the Interactive Tree of Life (iTOL web server version 5) [17], where branch coloring and clade annotations were applied to distinguish MT-A70, MTA70-like, and MTB clades. This analysis provided the evolutionary framework for subsequent gene structure, motif, and structural comparisons. Species were selected to represent the major land plant lineages, including bryophytes (*Physcomitrium patens* and *Marchantia polymorpha*), monocots, and dicots. The dataset was expanded to 51 sequences from 15 lineages by including the lycophyte *Selaginella moellendorffii*, the charophyte alga *Klebsormidium nitens*, the green alga *Chlamydomonas reinhardtii*, and *Saccharomyces cerevisiae* (IME4) as a fungal outgroup to root the tree. Despite extensive searches, well-annotated MT-A70 family sequences could not be retrieved for gymnosperms or ferns, likely reflecting incomplete gene annotation in conifer and fern genomes. Inclusion of additional divergent sequences in preliminary analyses resulted in poorly resolved nodes and reduced bootstrap support at key internal branches, necessitating the use of a curated representative set. This approach prioritizes phylogenetic clarity over taxonomic breadth and follows precedents in gene family analyses where alignment quality directly determines tree reliability [18].

### 2.3. Codon-Based Selection Analysis

To assess selective pressures acting on MTA70 coding sequences, FUBAR (Fast Unconstrained Bayesian AppRoximation) analysis was performed on the Datamonkey web server (datamonkey.org). For MTA70, unaligned CDSs for 12 taxa were submitted; internal alignment was performed by the server prior to analysis. For MTB, analysis was performed on the conserved C-terminal MT-A70-like domain (330 aa, corresponding to the full-length AT4G09980/AtMTB sequence) from 9 taxa, enabling robust codon alignment across this functionally conserved region. The universal genetic code was applied. Sites under significant purifying selection were identified at a posterior probability threshold of *p* ≥ 0.9. Full-length MTB sequences could not be reliably aligned for codon-based analysis due to high sequence length variation (990–4557 nt across taxa) and divergence in the N-terminal intrinsically disordered regions, which introduced alignment artifacts incompatible with FUBAR. Phylogenetic trees inferred by Datamonkey are provided in Supplementary Figures S1 and S2.

### 2.4. Gene Structure Analysis

To determine whether evolutionary divergence is reflected at the genomic level, comparative gene structure analysis was performed using TBtools II [19]. Genomic FASTA files, coding sequences (CDS), and corresponding GFF3 annotation files were obtained from the same database sources to ensure reliability. Exon–intron organization, untranslated regions (UTRs), and gene lengths were visualized using the Gene Structure View (Advanced) function in TBtools II. Exons (CDS), introns, and UTRs were displayed proportionally to scale in kilobases (kb), enabling direct comparison of gene architecture across different plant species and gene families.

### 2.5. Conserved Domain and Motif Analysis

To further examine whether sequence divergence is associated with functional differences, conserved protein domains within MT-A70 and MTA70-like/MTB proteins were identified using the NCBI Conserved Domain Database (CDD) via the Batch CD-Search tool [20,21]. Protein sequences were queried using default parameters with an E-value cutoff of 0.01. Domain architectures, including domain positions and boundaries, were extracted and visualized using the Simple BioSequence Viewer function in TBtools II, with proteins arranged according to their phylogenetic relationships. Detailed domain annotations of representative protein sequences are provided in Supplementary Table S1.

To identify conserved sequence features and assess divergence between protein groups, motif analysis was performed using MEME Suite version 5.4.1 [22]. Protein sequences were analyzed under the following parameters: motif discovery mode set to classic, maximum number of motifs set to 8, minimum motif width of 6 amino acids, and maximum motif width of 50 amino acids. All other parameters were kept at default settings. Identified motifs were mapped onto protein sequences and compared across MT-A70 and MTA70-like/MTB groups to assess conservation and divergence patterns. Motif position files generated by MEME were visualized using TBtools II.

To examine residue-level conservation within identified motifs, sequence logos were generated using WebLogo3 [23], which represents amino acid conservation based on information content measured in bits.

### 2.6. Promoter Cis-Regulatory Element Analysis

To investigate potential regulatory differences between MT-A70 family members, promoter regions were analyzed for *cis*-regulatory elements. Promoter sequences corresponding to 1500 bp upstream of the translation start codon (ATG) were extracted from genomic sequences obtained from Ensembl Plants (release 63). This window encompasses the proximal promoter region, which in Arabidopsis contains approximately 86% of transcription factor binding sites within −1000 bp to +200 bp of the transcription start site [24], and is in agreement with the sequence length used for development and validation of the PlantCARE database [25]. Extension to longer upstream regions introduces distal intergenic sequences with substantially reduced cross-species conservation, which would increase noise in comparative analyses across the phylogenetically diverse species examined here. *Cis*-regulatory element prediction was performed using the PlantCARE database [25], which identifies plant regulatory motifs based on position-specific scoring matrices derived from experimentally validated *cis*-elements.

To reduce redundancy from degenerate motif matches and enrich for high-confidence predictions, only *cis*-elements with a PlantCARE matrix score ≥ 8 were retained for quantitative analysis, and this threshold was applied uniformly across all categories. Identified cis-elements were classified according to their associated biological functions, including phytohormone responsiveness (auxin, abscisic acid, gibberellin, salicylic acid, jasmonic acid, and ethylene), abiotic stress responses (drought, low temperature, and light), biotic stress responses, and developmental regulation (meristem expression, endosperm expression, and circadian control). The frequency and distribution of *cis*-elements were quantified and visualized as bar plots. To assess whether differences in *cis*-element frequencies between MTA70 and MTB promoters were statistically significant, Mann–Whitney U tests were applied across 12 species per group using Python (SciPy v1.11). Two-sided tests were used for all comparisons, with significance threshold set at *p* < 0.05. A complete list of identified *cis*-elements is provided in Supplementary Table S2. Detailed distributions across individual species are provided in Supplementary Figure S3.

### 2.7. Expression Analysis and Heat Map Visualization

Expression values were extracted across multiple tissues and developmental stages (Supplementary Table S3). To enable cross-dataset comparison, raw expression values (TPM or FPKM) were log_2_-transformed after adding a pseudocount of 1 [log_2_ (TPM + 1)] to avoid undefined values for genes with zero expression and heatmaps were generated to visualize relative expression patterns across tissues and developmental stages, enabling comparison of regulatory profiles between gene groups. For ABA-responsive expression analysis, publicly available RNA-seq datasets from multiple plant species were retrieved from GEO, RiceXPro, and species-specific expression databases. Log2 fold change values under ABA treatment relative to control conditions were extracted and compiled for representative MTA70 and MTB homologs (Supplementary Table S4).

To further explore the potential biological roles of MT-A70 family genes, expression profiles were analyzed using publicly available transcriptomic datasets [13,26,27,28,29]. Data sources included Phytozome, the EMBL-EBI Expression Atlas, and the Bio-Analytic Resource (BAR) Plant eFP Browser as well as TENOR for tobacco expression data, and RiceXPro for rice expression data.

### 2.8. Structural Prediction and Protein–Protein Docking

To determine whether sequence divergence translates into structural differences, three-dimensional models of *Arabidopsis thaliana* MTA70 and MTB were obtained from the AlphaFold Protein Structure Database through UniProt entries for arabidopsis MTA70 (UniProt: O82486; AlphaFold model AF-O82486-F1) and MTB (UniProt: Q94A14; AlphaFold model AF-Q94A14-F1) [14]. AlphaFold models provide per-residue confidence scores (pLDDT), which were used to assess local model quality prior to downstream analysis. Regions with pLDDT values greater than 70 were considered sufficiently reliable for docking and interface characterization. The structures were inspected and visualized using UCSF ChimeraX (v1.11) before docking.

To further assess whether structural differences influence protein–protein interactions, docking analysis between Arabidopsis MTA70 and MTB was performed using the HADDOCK 2.2 web server [30]. Active residues defining the interaction interface were selected based on conserved surface-exposed residues detected from MEME motif analysis and evolutionary conservation mapping. Passive residues were automatically defined by HADDOCK as solvent-accessible residues surrounding the active residues within a 6.5 Å cutoff.

Docking was performed using standard HADDOCK parameters, with 1000 structures generated during the rigid-body docking stage, 200 structures refined during the semi-flexible simulated annealing stage, and 200 structures subjected to final explicit solvent refinement. Resulting structures were clustered using a 7.5 Å RMSD cutoff and ranked based on the HADDOCK score, which represents a weighted combination of van der Waals energy, electrostatic energy, desolvation energy, and restraint violation terms. The top-ranked cluster was selected for subsequent interface analysis.

To validate the HADDOCK-predicted interface without prior residue specification, independent blind docking was performed using the HDOCK web server (hdock.phys.hust.edu.cn). Full-length AlphaFold2 models of AtMTA70 (AF-O82486-F1) and AtMTB (AF-Q94A14-F1) were submitted without specifying any binding site residues. The top-ranked model (Model 1) and third-ranked model (Model 3) were selected for interface comparison.

Intermolecular contacts, including hydrogen bonds and salt bridges, were identified and measured using UCSF ChimeraX [31], with distance cutoffs of 3.5 Å for hydrogen bonds and 4.0 Å for electrostatic interactions. Structural conservation of predicted MTA70 and MTB models was assessed using the ESPript 3.0 server, which generates annotated sequence-structure alignments by comparing query structures against homologous entries in the SwissProt and AlphaFold databases. Secondary structure elements were extracted from AlphaFold2 models and mapped onto multiple sequence alignments to identify structurally conserved regions across MT-A70 family members.

### 2.9. Binding Affinity Prediction

To quantify the strength of the predicted interaction, binding free energy (ΔG) and dissociation constant (Kd) were estimated using the PRODIGY web server, which employs a contact-based statistical model trained on experimentally characterized protein–protein complexes [32]. The top-ranked HADDOCK docking model was submitted to PRODIGY as a single PDB file. Calculations were performed at a temperature of 25 °C using default server parameters.

Interface contacts were classified based on amino acid properties, including charged–charged, charged–polar, charged–apolar, polar–polar, polar–apolar, and apolar–apolar interactions. The non-interacting surface (NIS) composition was also recorded together with the predicted ΔG and Kd values. The predicted binding affinity was interpreted in the context of reported dissociation constant ranges for regulatory subunit interactions in RNA modification complexes.

### 2.10. Computational Alanine Scanning Mutagenesis

To identify residues predicted to contribute most significantly to MTA70–MTB interface stability, computational alanine scanning was performed using mCSM-PPI2 [33]. This approach systematically evaluates the energetic contribution of individual interface residues by calculating the change in binding free energy upon alanine substitution, thereby providing a ranked set of predicted hotspot residues as targets for future experimental mutagenesis. Interface residues revealed from the top-ranked HADDOCK docking model were systematically mutated to alanine in silico. The resulting change in binding free energy (ΔΔG, kcal/mol) was calculated for each mutation, where negative ΔΔG values indicate destabilization of the protein–protein interaction. Residues were classified as strongly destabilizing (ΔΔG ≤ −1.0 kcal/mol) or moderately destabilizing (−1.0 < ΔΔG ≤ −0.5 kcal/mol). The most significant residues were summarized in Table 1. mCSM-PPI2 provides point estimates of ΔΔG without formal confidence intervals, as is standard for machine learning-based structure energy prediction tools. Values should therefore be interpreted as relative rankings of destabilizing effect rather than precise thermodynamic measurements. Experimental validation via isothermal titration calorimetry or surface plasmon resonance will be required to confirm the predicted energetic contributions.

### 2.11. Gene Ontology Enrichment Analysis of the AtMTB Co-Expression Network

To investigate functional context of the MTB gene family, genes co-expressed with *AtMTB* (AT4G09980) in *Arabidopsis thaliana* were analyzed using the ATTED-II co-expression database, which integrates publicly available microarray and RNA-seq datasets to generate genome-wide co-expression networks [34]. The top 50 co-expressed genes ranked by mutual rank were retrieved and used for Gene Ontology (GO) enrichment analysis.

GO enrichment analysis was performed using the g:Profiler web application, with the Arabidopsis thaliana genome used as the statistical background and the Benjamini–Hochberg method applied to control the false discovery rate (FDR). Significantly enriched GO terms, including Biological Process (GO:BP), Molecular Function (GO:MF), and Cellular Component (GO:CC), were identified using an adjusted *p*-value threshold of FDR < 0.05.

The top enriched terms from each ontology category were selected for visualization and represented as bubble plots, where bubble size indicates the number of genes associated with each term and color represents the −log_10_ transformed FDR-adjusted *p*-value, with warmer colors indicating higher statistical significance. Visualization was performed using the SRplot online tool.

## 3. Results

### 3.1. Phylogenetic Relationships of MT-A70 Family Proteins Across Land Plants

**Figure 1:**
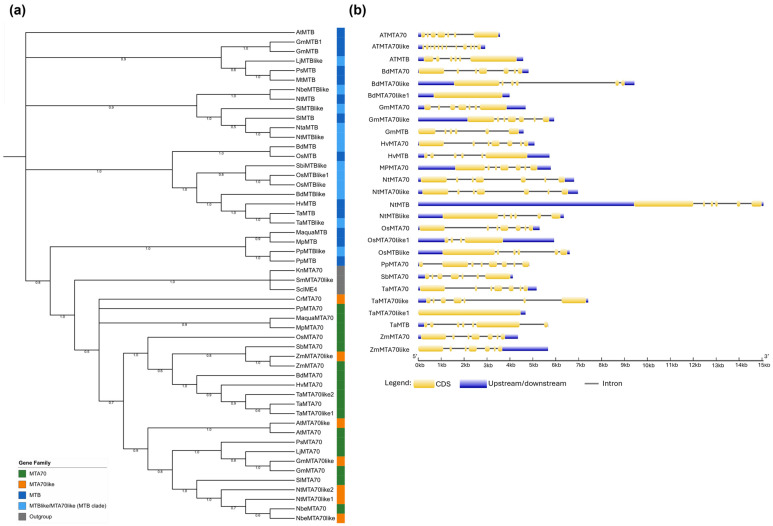
Phylogenetic relationships and gene structure organization of MT-A70 family proteins across representative species. (**a**) Maximum likelihood phylogenetic tree constructed using full-length MTA70, MTB, and MTA70-like protein sequences from 15 representative species. Multiple sequence alignment was performed using MUSCLE and the tree was inferred under the Jones-Taylor-Thornton (JTT) substitution model with 1000 bootstrap replicates. Bootstrap support values of 70% or greater are shown at internal nodes. Colored strips adjacent to the tree indicate clade membership: green, MTA70; blue, MTB; orange, MTA70-like/MTBlike. (**b**) Exon-intron organization of MT-A70 family genes displayed in the same order as the phylogenetic tree. Yellow boxes represent coding sequences (CDS), blue boxes represent upstream and downstream regions, and gray lines represent introns, as indicated in the legend. Gene structure analysis reveals conserved exon-intron architecture within the MTA70 clade and extensive structural divergence within MTB and MTA70-like members across plant lineages.

To investigate the evolutionary relationships within the plant MT-A70 family, we constructed a maximum likelihood phylogenetic tree. The analysis was based on 51 full-length protein sequences from 15 representative species, including newly added lycophyte (*Selaginella moellendorffii*, SmMTA70like) and charophyte alga (*Klebsormidium nitens*, KnMTA70) sequences. The phylogenetic tree resolved three strongly supported monophyletic groups corresponding to MTA70, MTA70-like, and MTB proteins. Most major nodes were supported by bootstrap values exceeding 85% (Figure 1a). Canonical MTA70 proteins formed a compact and highly conserved clade with relatively short branch lengths. Within this group, angiosperm sequences separated into monocot and dicot subclades, consistent with known species relationships. Sequences from *P. patens* and *M. polymorpha* clustered within the MTA70 clade rather than forming an outgroup, indicating that canonical MTA70 proteins were already present at the base of land plant evolution (Figure 1a). Importantly, *Selaginella moellendorffii* and *Klebsormidium nitens* sequences clustered at the base of the MTA70 clade with strong bootstrap support (100%), confirming that the phylogenetic framework extends to lycophytes and charophyte algae and that the MTA70 lineage predates the divergence of vascular plants. Bryophyte sequences were positioned within the MTA70 clade rather than forming a separate sister group, and together with the short branch lengths observed, this pattern is consistent with selective constraint at functionally critical residues rather than a methodological artifact such as long-branch attraction [18].

In contrast, MTB proteins formed a clearly distinct clade separated from canonical MTA70 with strong bootstrap support. This group exhibited longer branch lengths, reflecting greater sequence divergence. The expanded dataset of 51 sequences included fungal (ScIME4), green algal (CrMTA70), charophyte (KnMTA70), and lycophyte (SmMTA70like) outgroups, which correctly rooted the tree outside all land plant sequences with bootstrap support of 1.00. MTB sequences from both bryophytes were located within this clade, indicating that the divergence of MTB from canonical MTA70 predates the separation of major land plant lineages. MTA70-like proteins constituted a third clade, situated between the established MTA70 and MTB groups, displaying moderate bootstrap support. This intermediate positioning is consistent with MTA70-like proteins sharing characteristics of both lineages. This phylogenetic pattern supports an early separation of catalytic and non-catalytic components within the plant MT-A70 family. FUBAR analysis of MTA70 coding sequences (810 sites, 12 taxa) identified 2 sites under significant purifying selection at posterior probability *p* ≥ 0.9 (Site 1: *p* = 0.951; Site 20: *p* = 0.914), and 2 sites under positive selection (*p* ≥ 0.9), with no sites reaching *p* ≥ 0.95 for either category (Supplementary Table S5, Figure S4). The concentration of selective constraint at specific functional sites, rather than genome-wide, is in agreement with the evolutionary pattern of enzymatic subunits in which catalytic residues are selectively constrained while flanking regions tolerate greater divergence. FUBAR analysis of the MTB C-terminal MT-A70-like domain (330 sites, 9 taxa) revealed substantially stronger purifying selection compared to MTA70, with 37 sites under significant purifying selection at *p* ≥ 0.9 (including 11 sites at *p* ≥ 0.95) and no sites under positive selection (Supplementary Table S5, Figure S5). The mean β/α ratio of 0.146 and the proportion of sites with β < α (97.0%) indicate pervasive selective constraint across the MT-A70 scaffold domain of MTB, consistent with its essential role in complex architecture and RNA substrate positioning.

**Figure 2:**
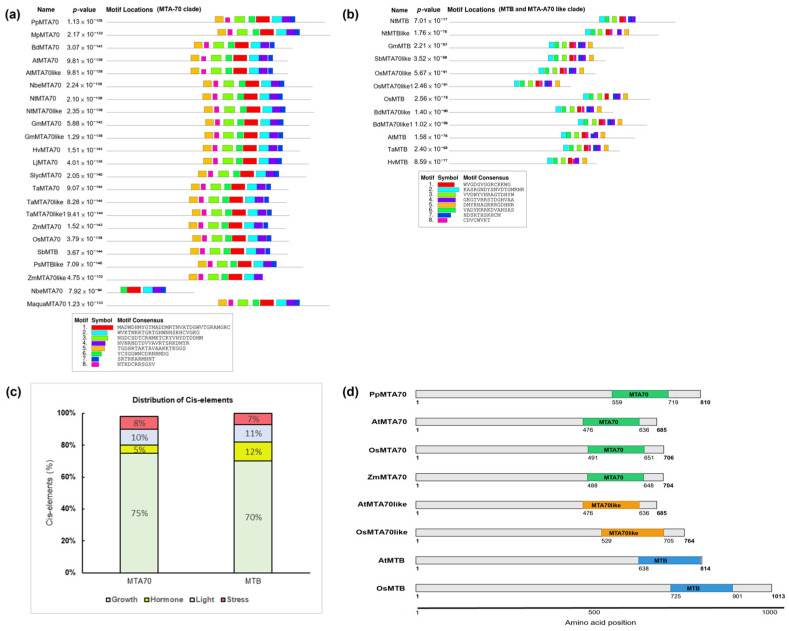
Conserved motif architecture and domain organization of MTA70 family proteins. (**a**) Conserved motif distribution in representative members of the MTA70 clade. Motifs were identified using MEME and are depicted as colored boxes. Proteins within this clade exhibit highly conserved motif composition and arrangement, reflecting functional conservation. Corresponding motif consensus sequences and statistical significance (*p*-values) are indicated. (**b**) Conserved motif distribution in MTB and MTA70-like clades. Compared to the MTA70 clade, these proteins display distinct and more variable motif patterns, suggesting functional divergence. (**c**) Percentage distribution of *cis*-acting regulatory elements in MTA70 and MTB promoters, showing enrichment of phytohormone-responsive elements in MTB genes. (**d**) Schematic representation of domain organization of selected representative proteins. The conserved MT-A70 domain (green), MT-A70-like region (orange), and MTB domain (blue) are shown relative to full-length protein sequences. Amino acid positions are indicated, highlighting conserved domain placement in MTA70 proteins and the extended C-terminal localization characteristic of MTB proteins. Detailed *cis*-element composition across individual species is shown in Supplementary Figure S3 and Table S2.

The MTA70-like clade occupied a phylogenetically intermediate position between canonical MTA70 and MTB, with moderate bootstrap support, though the functional significance of this positioning remains unclear in the absence of biochemical or genetic data (Figure 1a). Motif analysis revealed that MTA70-like proteins share some, but not all, of the conserved catalytic motifs present in MTA70, while also exhibiting MTB-like divergence in surface-exposed regions (Figure 2a,b). The DPPW/DPPY catalytic motif, invariant across MTA70 sequences, was absent or partially substituted in MTA70-like proteins, suggesting partial erosion of catalytic capacity. Domain architecture analysis further showed that MTA70-like proteins retain an MT-A70-related fold but with variable C-terminal extensions not observed in canonical MTA70. Expression profiling indicated that MTA70-like transcripts are expressed at lower levels than either MTA70 or MTB across most tissues examined (Supplementary Figure S6; expression patterns across six plant species). Together, these features suggest that the MTA70-like clade may represent an evolutionary intermediate that has partially decoupled from the catalytic constraints of MTA70 but has not yet acquired the full regulatory specialization of MTB, a hypothesis that warrants future functional investigation.

### 3.2. Gene Structure and Domain Architecture of MTA70 and MTB Proteins

To determine whether the divergence between MTA70 and MTB is reflected at the genomic and protein levels, we compared exon–intron organization and domain architecture across representative species (Figure 1b and Figure 2d). Canonical MTA70 genes showed a conserved multi-exon structure across all examined species. Although total gene length and intron size varied, the number and relative arrangement of coding exons remained largely consistent from bryophytes to angiosperms (Figure 1b). MTA70 genes from *P. patens* and *M. polymorpha* displayed simpler architectures with fewer and shorter introns, whereas angiosperm homologs contained longer intronic regions. Despite these differences, the overall exon organization was preserved across lineages.

Domain analysis further revealed that MTA70 proteins uniformly contain a single, well-defined MT-A70 domain spanning most of the protein length, with the SAM-binding region located toward the C-terminus (Figure 2d). This domain organization was conserved across all species examined (Supplementary Table S1).

In contrast, MTB genes displayed substantial variation in exon–intron structure (Figure 1b). The number of introns ranged from three to more than twelve across species, and intron lengths varied considerably. Differences in exon arrangement and untranslated regions were also observed among closely related species. Domain architecture of MTB proteins was more variable compared to MTA70 (Figure 2d). While MTB proteins retained an MT-A70-related domain or fragment, this domain was often truncated or divergent. In addition, MTB proteins contained variable accessory domains, with domain composition differing across species and paralogs. Overall, MTA70 genes maintain a conserved genomic and domain organization, whereas MTB genes exhibit substantial structural variability across land plant lineages.

### 3.3. Conserved Motif Composition and Residue-Level Variation in MTA70 and MTB Proteins

To assess whether the structural differences between MTA70 and MTB are reflected at the sequence level, we analyzed conserved motifs using MEME and examined residue conservation using WebLogo (Figure 2a, Supplementary Figure S7). MTA70 proteins contained several highly conserved motifs that were steadily detected across all examined species, including bryophytes, monocots, and dicots (Figure 2a). The relative positions of these motifs were strongly conserved, with minimal variation in spacing. The characteristic DPPW/DPPY motif was present in all MTA70 sequences at conserved positions [8,35]. WebLogo analysis displayed strong residue conservation at positions corresponding to predicted SAM-binding and catalytic regions, with high information content across key sites (Supplementary Figure S7a).

In contrast, MTB proteins displayed a distinct motif composition (Figure 2b). Although conserved motifs were identified, their sequences and spatial arrangement differed from those in MTA70. Residues that were highly conserved in MTA70 were frequently substituted or absent in MTB proteins, and the DPPW/DPPY motif was not detected. WebLogo analysis revealed reduced conservation at positions corresponding to the catalytic core, with increased variability across aligned sequences (Supplementary Figure S7b). This reduction in information content across key positions highlights the loss of catalytic residue conservation in MTB proteins.

Conserved motifs in MTB proteins were distributed across regions predicted to be surface-exposed rather than clustered within a defined catalytic core. Several MTB-specific motifs showed moderate conservation despite overall sequence divergence. MTA70 proteins retain conserved catalytic motifs across land plants, whereas MTB proteins harbor distinct motif patterns and reduced residue-level conservation, consistent with the loss of canonical catalytic signatures.

### 3.4. Structural Modeling Reveals Distinct Architectures of Arabidopsis MTA70 and MTB

**Figure 3:**
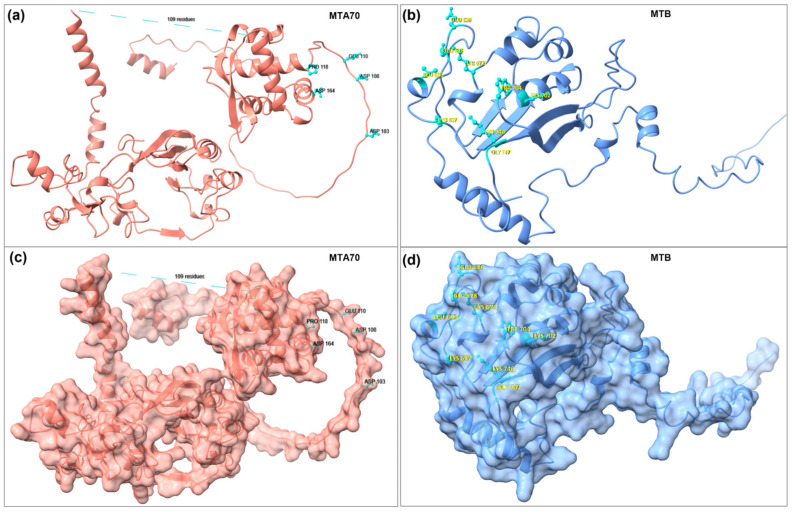
Structural models of MTA70 and MTB proteins and their predicted interacting residues. (**a**) Ribbon representation of MTA70 (salmon) showing the overall protein architecture predicted by AlphaFold2. The dashed line indicates 109 low-confidence N-terminal residues (pLDDT < 70) excluded prior to docking preparation. Predicted interface residues (Asp103, Asp108, Glu110, Gly116, Pro118, and Asp164) are highlighted in cyan stick representation. (**b**) Ribbon representation of MTB (blue) displaying the structured interaction domain. Predicted interface residues (Glu626, Glu628, Leu633, Lys637, Lys673, Lys702, Trp704, Lys746, and Gly747) are shown in cyan stick representation, distributed across a broad surface-exposed region consistent with a non-catalytic scaffold architecture. (**c**) Surface representation of AtMTA70 highlighting the accessible distribution of predicted interface residues (cyan) across the protein surface. (**d**) Surface representation of MTB showing the predicted interaction residues (cyan) concentrated within a well-defined surface-exposed region. Interface residues in MTB showed very high structural confidence (pLDDT range 90.1–98.4). Among AtMTA70 interface residues, Pro118 and Asp164 indicated high confidence (pLDDT 89.6 and 89.9, respectively), while Asp103, Asp108, Glu110, and Gly116 fall within low-confidence regions (pLDDT 29.6, 27.6, 30.8, and 61.9, respectively) and should be interpreted with caution pending experimental validation.

To determine whether sequence and motif differences translate into structural divergence, we generated three-dimensional models of Arabidopsis MTA70 and MTB (Figure 3). The MTA70 model adopted the characteristic fold of SAM-dependent RNA methyltransferases, with a compact and globular architecture organized around a central beta-sheet flanked by alpha-helices (Figure 3a,c). Conserved motifs identified in sequence analysis mapped to a well-defined catalytic pocket within the core of the protein. This pocket forms a spatially clustered region corresponding to predicted SAM-binding and catalytic residues.

In contrast, the MTB model displayed a more extended and less compact architecture (Figure 3b,d). Although it retained an overall MT-A70-related fold, the structure lacked a defined catalytic pocket. Residues corresponding to conserved catalytic motifs in MTA70 were absent or substituted in MTB, and no comparable active site geometry was observed. Interface residues in MTB (Glu626, Glu628, Leu633, Lys637, Lys673, Lys702, Trp704, Lys746, and Gly747) showed very high structural confidence in the AlphaFold model (pLDDT range 90.1–98.4). Among AtMTA70 interface residues, Pro118 and Asp164 displayed high confidence (pLDDT 89.6 and 89.9, respectively), whereas Asp103, Asp108, Glu110, and Gly116 fall within low-confidence regions (pLDDT 29.6, 27.6, 30.8, and 61.9, respectively), indicating that these MTA70 interface contacts should be interpreted with caution and await experimental validation. Structural comparison of the docked AtMTA70–MTB complex against the SwissProt and AlphaFold databases using ENDscript confirmed that the predicted interface residues map to structurally conserved positions across homologous MT-A70 family proteins (Supplementary Figures S8 and S9). Surface representation of MTB revealed broad, accessible regions with distributed conserved residues, rather than a confined catalytic cavity. These architectural features are characterized by broad surface exposure rather than a buried active site. The structural models show that MTA70 retains a compact catalytic fold, whereas MTB adopts an extended structure lacking a defined catalytic pocket and enriched in surface-exposed regions.

### 3.5. Docking Analysis of the MTA70–MTB Complex Identifies a Defined Interaction Interface

**Figure 4:**
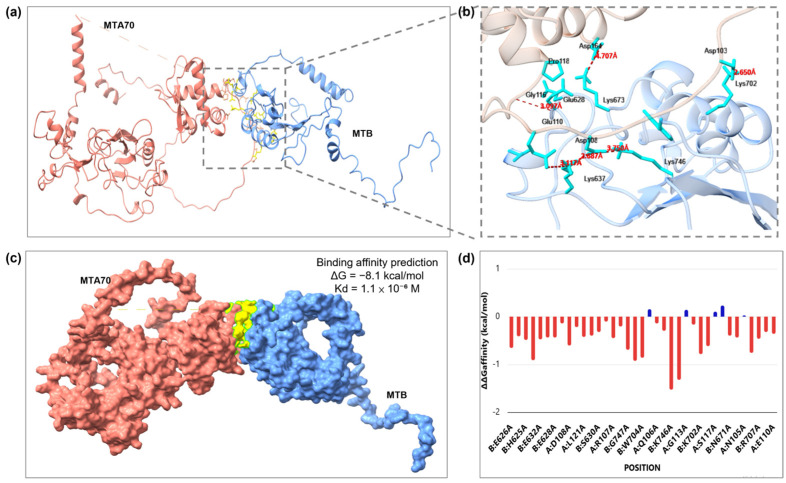
Structural model, interaction interface, and mutational analysis of the Arabidopsis MTA70–MTB protein complex. (**a**) Cartoon representation of the docked complex predicted using HADDOCK, showing MTA70 (salmon) and MTB (blue). The predicted interaction interface is highlighted by a dashed box, with interface residues shown in yellow stick representation. (**b**) Enlarged view of the interaction interface showing key intermolecular contacts. A central hydrogen bond is observed between Gly116 of MTA70 and Glu628 of MTB (3.097 Å). Salt bridges are formed between Asp108–Lys637 (2.687 Å), Glu110–Lys637 (3.117 Å), Asp108–Lys746 (3.750 Å), Asp103–Lys702 (2.650 Å), and an electrostatic contact between Asp164–Lys673 (4.707 Å), collectively contributing to stabilization of the complex. Interface residues from MTA70 (salmon ribbon) and MTB (blue ribbon) are shown in cyan stick representation with red dashed lines indicating intermolecular contacts. (**c**) Surface representation of the docked complex showing MTA70 (salmon) and MTB (blue) with the predicted binding interface highlighted in yellow, emphasizing the spatial complementarity between the two proteins. The predicted binding affinity of the complex is ΔG = −8.1 kcal/mol with a dissociation constant (Kd) of 1.1 × 10^−6^ M, as estimated by PRODIGY. (**d**) Computational alanine scanning mutagenesis analysis of interface residues performed using mCSM-PPI2, showing the effect of individual substitutions on binding free energy (ΔΔG, kcal/mol). Destabilizing mutations are shown as red bars and minor stabilizing substitutions as blue bars. Residues Lys746 and Lys637 of MTB exhibited the strongest destabilizing effects (ΔΔG ≤ −1.0 kcal/mol), identifying them as critical hotspots for complex stability.

To characterize the interaction between MTA70 and MTB, we performed protein–protein docking using HADDOCK, guided by conserved surface residues identified from sequence analysis (Figure 4). The top-ranked docking cluster predicted a stable heterodimeric complex with a HADDOCK score of −73.7 ± 3.1 and a Z-score of −1.8. The cluster demonstrated good convergence, with low structural deviation among solutions, indicating a stable binding mode. Binding affinity estimation using PRODIGY yielded a predicted free energy of −8.1 kcal/mol and a dissociation constant in the low micromolar range. The HADDOCK binding energy of −281.28 kcal/mol further supports complex stability, with the electrostatic contribution (−367.7 kcal/mol) substantially exceeding the van der Waals term (−25.8 kcal/mol), indicating that the interface is predominantly electrostatically driven.

The predicted interface is located at the junction between the MTA70 catalytic core and the extended surface of MTB (Figure 4a,b). A central hydrogen bond was found between Gly116 of MTA70 and Glu628 of MTB (3.097 Å), supported by salt bridges between Asp108–Lys637 (2.687 Å), Asp103–Lys702 (2.650 Å), Glu110–Lys637 (3.117 Å), Asp108–Lys746 (3.750 Å), and an electrostatic contact between Asp164–Lys673 (4.707 Å), collectively stabilizing the interaction interface. No AIR violations were observed, further supporting a well-defined and energetically favorable binding mode (Figure 4c). However, it should be noted that among the AtMTA70 residues contributing these contacts, Asp103, Asp108, Glu110, and Gly116 fall within low-confidence regions of the AlphaFold model (pLDDT 29.6, 27.6, 30.8, and 61.9, respectively), and the predicted contacts involving these residues should therefore be interpreted with caution pending experimental structural validation. Structural alignment using ESPript 3.0 confirmed that the predicted interface residues of AtMTA70 and AtMTB map to structurally conserved positions in homologous MT-A70 family proteins across diverse organisms (Supplementary Figures S8 and S9).

The interaction surface spans approximately 1435 Å^2^, a value within the range typically observed for obligate heterodimeric protein–protein complexes (900–2000 Å^2^), consistent with a stable and specific interaction [36] and involves a network of polar and charged residues distributed across both proteins. Computational alanine scanning revealed several residues contributing to interface stability (Table 1; Figure 4d). Residues Lys746 and Lys637 of MTB showed the strongest destabilizing effects upon mutation (ΔΔG ≤ −1.0 kcal/mol), indicating their critical role in maintaining complex stability. Additional residues, including Pro118 of MTA70 and Trp704, Lys702, Leu633, Gly747, Glu626, and Lys673 of MTB, showed moderate destabilizing effects, suggesting that the interaction interface is supported by a distributed network of energetically significant contacts rather than a single dominant hotspot. The agreement between the graphical representation of mutation effects (Figure 4d) and quantitative ΔΔG values (Table 1) further supports the reliability of the predicted interaction interface.

To independently validate the HADDOCK-predicted interface, blind docking was performed using HDOCK without specifying any binding site residues. The top-ranked HDOCK model yielded a docking score of −327.00 and confidence score of 0.9718, indicating high-confidence placement (Supplementary Figure S10). Three-dimensional distance analysis of interface residues revealed strong convergence on the MTB interaction surface: Leu633 was identified identically by both HADDOCK and HDOCK (0.0 Å), while Glu626 and Lys637 were within 4 Å of adjacent HDOCK-predicted contacts, and Lys673, Lys746, and Gly747 within 6 Å. Both methods consistently revealed the C-terminal domain of MTB (residues 612–757) as the primary interaction surface. The HDOCK result was reproducible across top-ranked models, with 37 shared receptor residues and 31 shared ligand residues between Model 1 and Model 3, confirming internal consistency of the blind docking prediction (Table S6).

### 3.6. Promoter Architecture, Expression Patterns, and Functional Association of MTB Genes

To examine whether functional divergence between MTA70 and MTB extends to transcriptional regulation, we analyzed promoter architecture, expression patterns, and co-expression networks of MTB. Promoter analysis revealed distinct regulatory patterns between MTA70 and MTB genes (Figure 2c, Supplementary Figure S2). Statistical comparison of *cis*-element distributions across 12 species per group showed that TGACG-motif, a MeJA/jasmonate-responsive element, was significantly enriched in MTB promoters compared to MTA70 (mean 2.58 vs. 0.67; Mann–Whitney U, *p* = 0.025). Combined jasmonate-responsive elements (CGTCA + TGACG) showed consistent enrichment in MTB (mean 4.00 vs. 1.50; *p* = 0.026). ABRE revealed a 3.4-fold higher count in MTB promoters (total 62 vs. 18), though this did not reach statistical significance (*p* = 0.307). Core promoter elements (CAAT-box, TATA-box) and stress-responsive elements (ARE, MBS) were uniformly distributed between groups (*p* > 0.27), in line with conserved transcriptional machinery (Supplementary Table S7).

MTA70 promoters were enriched in elements associated with growth and development, including TATA-box, CAAT-box, and circadian-related motifs [37] and showed relatively similar composition across species. In contrast, MTB promoters contained a higher abundance and diversity of hormone-responsive, light-responsive, and stress-related elements, including ABRE, AuxRR-core, and jasmonate- and salicylic acid-responsive motifs. The composition of these elements varied across species, indicating greater regulatory diversity in MTB promoters.

Expression profiling across six plant species (Supplementary Figure S6 and Table S3) showed that MTA70 homologs are broadly expressed across tissues and developmental stages. In Arabidopsis, *AtMTA70* transcripts were detected in seeds, vegetative tissues, and reproductive organs. Similar expression patterns were observed in soybean, wheat, and rice, where MTA70 homologs showed uniform expression across multiple organs. In contrast, MTB transcripts displayed more variable expression patterns with preferential accumulation in reproductive and embryogenic tissues. In Arabidopsis, *AtMTB* expression was elevated in seeds and floral tissues relative to vegetative organs. Similar enrichment was observed in soybean, wheat, and rice, where MTB homologs showed higher expression in reproductive structures such as flowers, embryos, and inflorescences. This pattern was also observed in bryophytes, where MTB transcripts were more abundant in reproductive structures compared to other tissues.

**Figure 5:**
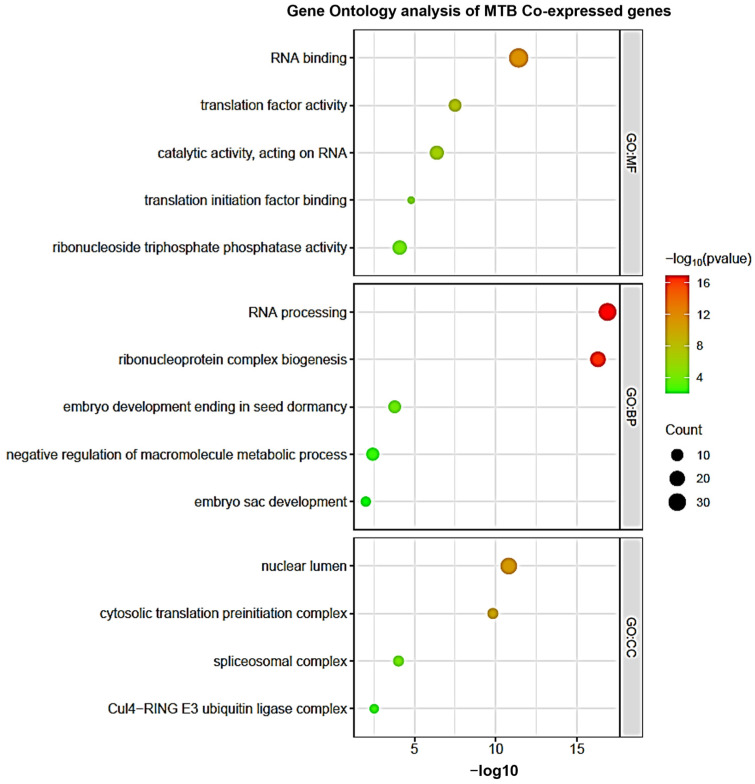
Functional enrichment and expression profiling of MTB co-expressed genes. Gene Ontology (GO) enrichment analysis of MTB co-expressed genes identified from ATTED-II and analyzed using g:Profiler. The top enriched terms are shown for molecular function (MF), biological process (BP), and cellular component (CC), highlighting RNA binding, RNA processing, and ribonucleoprotein complex biogenesis, consistent with a broader role of MTB in RNA metabolism beyond its function in the m6A writer complex.

Gene Ontology analysis of genes co-expressed with *AtMTB* revealed significant enrichment in RNA-related processes (Figure 5), though direct functional links between MTB and these processes remain to be experimentally confirmed. The most enriched Biological Process terms included RNA processing and ribonucleoprotein complex biogenesis. Molecular Function terms were dominated by RNA binding and translation-related activities, while Cellular Component terms highlighted nuclear and ribonucleoprotein-associated compartments. MTA70 genes exhibit stable expression and conserved promoter architecture, whereas MTB genes display greater regulatory diversity, tissue-specific expression, and association with RNA-related functional networks. Taken together, promoter composition, expression profiles, and ABA-responsive transcriptional changes are consistent with the interpretation that MTB genes are subject to complex regulatory control associated with developmental and stress-responsive pathways. The enrichment of ABRE (ABA-responsive element) motifs in MTB promoters, combined with ABA-responsive expression changes observed across species (Supplementary Table S4), raises the possibility that m6A deposition through the MTA70–MTB complex may be modulated under osmotic or drought stress conditions. This is in agreement with emerging evidence that epitranscriptomic regulation may be coupled to ABA signaling in plants [38,39].

## 4. Discussion

A key finding of this study is the remarkable conservation of MTA70 across land plant evolution. Evidence from phylogenetic analysis, gene structure, domain organization, and motif conservation indicates that MTA70 has remained highly constrained from bryophytes to angiosperms. This level of conservation is characteristic of enzymes that perform essential biochemical functions, where even minor perturbations can disrupt catalytic activity. The developmental arrest observed in *Atmta* mutants at the globular embryo stage underscores this constraint and highlights the indispensable role of m6A deposition in plant development [2]. The depth of this conservation is particularly notable given the evolutionary distance spanned in this analysis. MTA70 proteins from bryophytes such as *M. polymorpha* and *P. patens* retain key catalytic motifs and structural features that are also present in angiosperms, suggesting that the core methyltransferase function was established early in land plant evolution and has been maintained under strong purifying selection. This pattern is consistent with general principles of gene family evolution, in which catalytic subunits of essential complexes display high sequence and structural conservation owing to functional constraints [9].

The conservation of MTA70 in plants also parallels the evolutionary stability of its functional counterpart in animals. In mammals, METTL3 serves as the catalytic core of the m6A writer complex and shows similar resistance to sequence divergence, reflecting the biochemical constraints imposed by methyltransferase activity [5,6]. The convergence of these patterns across kingdoms supports the notion that the catalytic component of the m6A writer complex is universally constrained by its enzymatic role, regardless of organismal context.

In contrast to the stability of MTA70, MTB proteins display substantial sequence and structural divergence across land plant lineages. This divergence is evident in their variable exon–intron organization, domain architecture, and motif composition, as well as in the absence of conserved catalytic residues. Such patterns are in line with the evolutionary behavior of non-catalytic subunits in multi-protein complexes, which often exhibit relaxed structural constraints and greater evolutionary flexibility [40,41,42]. Rather than maintaining catalytic function, these proteins typically evolve to support complex assembly, substrate recognition, or regulatory control.

The structural models generated in this study provide further insight into this functional divergence. While MTA70 retains a compact catalytic fold with a defined active site, MTB adopts an extended architecture lacking catalytic features but enriched in surface-exposed regions. Structural database comparison of the docked complex using ENDscript further supports this interpretation, as the predicted interface residues map to structurally conserved positions in homologous MT-A70 family proteins across both the SwissProt and AlphaFold databases (Supplementary Figures S8 and S9). This structural organization is consistent with a role in protein–protein interaction rather than enzymatic activity. A similar division of labor has been described in the mammalian METTL3–METTL14 complex, where METTL14 retains an MT-A70-like fold but functions primarily as a structural scaffold that enhances RNA binding and stabilizes the catalytic subunit [6,43]. Quantitative structural superposition using ChimeraX Matchmaker further supports this conservation: AtMTA70 superposed onto human METTL3 (AlphaFold model AF-Q86U44) with an RMSD of 0.333 Å over 121 pruned Cα atom pairs (alignment score 694.7), while AtMTB superposed onto human METTL14 (AlphaFold model AF-Q9HCE5) with an RMSD of 0.561 Å over 182 pruned Cα atom pairs (alignment score 716.8), confirming high structural conservation of both catalytic and non-catalytic MT-A70 fold subunits between Arabidopsis and human m6A writer complex components (Supplementary Table S8).

Together, these observations suggest a model in which MTA70 represents a highly conserved catalytic core, while MTB has diverged into a non-catalytic regulatory scaffold. The persistence of this division across land plant evolution indicates that it represents a fundamental organizational principle of the plant m6A writer complex.

The structural and docking analyses presented here provide a computationally derived predictive framework for understanding how MTB may function as a regulatory partner of MTA70 in plants. Subject to experimental validation, the predicted Arabidopsis MTA70–MTB complex is consistent with a stable heterodimeric interaction defined by a specific interface geometry, with a central hydrogen bond between Gly116 of MTA70 and Glu628 of MTB, supported by flanking electrostatic interactions involving Asp108–Lys637 and Asp164–Lys673. The convergence of these contacts within a confined interface region supports a well-defined binding orientation rather than nonspecific association. Nevertheless, it should be acknowledged that the MTA70 residues forming the central hydrogen bond (Gly116, pLDDT 61.9) and key salt bridge contacts (Asp103, Asp108, and Glu110, pLDDT 29.6–30.8) fall within low-confidence regions of the AlphaFold model, and the specific contacts predicted at these positions remain tentative until confirmed by experimental structural data such as cryo-EM or X-ray crystallography.

The energetic properties of the docked complex further support this interpretation. The favorable HADDOCK score, negative Z-score, and predicted binding affinity in the low micromolar range are indicative of stable yet reversible interactions typical of regulatory subunits within multi-protein complexes [30,32]. HADDOCK scores below −50 kcal/mol are generally indicative of high-quality docking solutions, and a Z-score of −1.8 places this cluster well outside the distribution of non-specific docking poses, further supporting the specificity of the predicted interface [44]. For comparison, the experimentally determined METTL3–METTL14 heterodimer, whose crystal structure is deposited in the PDB (PDB: 5IL2), exhibits a buried surface area of approximately 1600–1800 Å^2^ and is stabilized by an analogous network of polar and electrostatic contacts at the heterodimer junction [6,43]. The predicted MTA70–MTB interface area of 1435 Å^2^ is somewhat smaller than but comparable to this range, suggesting that the predicted plant complex possesses energetic and geometric properties comparable to the experimentally validated mammalian writer complex. Such interaction strengths are characteristic of protein pairs that must assemble efficiently while retaining the flexibility required for dynamic regulation.

Alanine scanning analysis revealed key residues contributing to interface stability. In particular, Lys746 and Lys637 of MTB showed strong destabilizing effects upon mutation, highlighting their importance in maintaining complex integrity (ΔΔG ≤ −1.0 kcal/mol; Table 1). The prominence of these residues on the MTB surface is consistent with its role as the primary interaction platform within the complex, while the more limited contribution from MTA70 reflects its compact catalytic architecture. The distributed nature of the interface, involving multiple residues with moderate destabilizing contributions (Table 1), suggests that complex stability arises from cooperative interactions rather than a single dominant hotspot. The concordance between the graphical representation of mutation effects (Figure 4d) and the quantitative ΔΔG values (Table 1) strengthens confidence in the predicted interaction interface and highlights key residues for future experimental validation.

The predicted interaction interface shows notable conceptual parallels with the mammalian METTL3–METTL14 complex. In that system, METTL14 forms a stable heterodimer with METTL3 through a network of polar and electrostatic interactions, positioning RNA substrates for efficient methylation [6,43]. Although sequence similarity between plant MTB and animal METTL14 is limited, quantitative structural superposition confirms a comparable fold (AtMTB vs. METTL14 RMSD = 0.561 Å; AtMTA70 vs. METTL3 RMSD = 0.333 Å), suggesting a conserved division of labor in which a non-catalytic MT-A70-related protein stabilizes and supports the catalytic subunit.

Importantly, the identification of specific interface residues provides a set of experimentally testable predictions. Targeted mutagenesis of Lys746 and Lys637 in MTB, followed by binding and functional assays, would allow direct evaluation of their roles in complex formation and m6A deposition. Such experiments will be essential for validating the structural model and for establishing the molecular basis of writer complex assembly in plants.

Observations from this study support a model in which MTB functions as a structurally specialized interaction scaffold that stabilizes MTA70 and contributes to the assembly of the plant m6A writer complex. Notably, the identification of multiple moderately destabilizing residues in addition to key hotspots supports the idea that the MTA70–MTB interaction is governed by a distributed electrostatic interface, a feature commonly observed in regulatory protein–protein complexes that require both stability and dynamic flexibility.

Beyond structural divergence, our results reveal a clear separation in the regulatory and expression profiles of MTA70 and MTB, pointing to distinct functional roles within the plant m6A writer system. MTA70 genes show relatively stable promoter architecture enriched in growth- and development-associated elements, together with broad expression across tissues and developmental stages. This pattern suggests a constitutive role in maintaining global m6A levels, as expected for a catalytic component required throughout the plant life cycle [2].

In contrast, MTB genes display a more complex and variable regulatory landscape. The enrichment of hormone-responsive, light-responsive, and stress-related *cis*-elements in MTB promoters suggests that their transcription is more sensitive to environmental and developmental cues. Such regulatory diversity suggests that non-catalytic subunits of multi-protein complexes frequently evolve flexible expression patterns that enable context-dependent modulation of core enzymatic activity [45]. This principle is well illustrated in other plant systems; for example, the MeJA-responsive transcription factor PgMYB2 in Panax ginseng coordinates hormone-responsive gene regulation through promoter-level *cis*-element recognition, demonstrating how phytohormone signals are transduced into transcriptional outputs via conserved regulatory modules [46]. The jasmonate-responsive enrichment in MTB promoters identified in this study (TGACG-motif, *p* = 0.025) suggests that MTB expression may be coordinated with JA-mediated stress and defense signaling in addition to ABA responses, extending the range of environmental inputs that may modulate m6A deposition in plants.

The expression data further support this distinction. Across all examined species, MTB transcripts show preferential accumulation in reproductive and embryogenic tissues, including seeds, flowers, and gametophytic structures. Notably, m6A demethylation by ALKBH10B has been shown to regulate Arabidopsis floral transition [47], and the higher abundance of MTB transcripts in floral organs indicates that the writer complex itself is subject to tissue-specific regulation during this critical developmental phase. The conservation of this expression pattern from bryophytes to angiosperms suggests that MTB-mediated regulation of m6A deposition is particularly important during reproductive development. These stages are characterized by rapid transcriptional reprogramming and tight developmental control, conditions under which precise post-transcriptional regulation is particularly critical [3,4].

Gene Ontology enrichment of the *AtMTB* co-expression network provides additional insight into its functional context. The strong enrichment of RNA processing and ribonucleoprotein complex-related terms indicates that MTB is associated with broader RNA regulatory pathways beyond the core writer complex. This observation is consistent with studies in animal systems showing that m6A deposition is functionally linked to RNA processing and splicing machinery [1].

This analysis reveals that MTB functions not only as a structural scaffold for MTA70 but also as a regulatory node that links m6A deposition to developmental and environmental signaling. By integrating transcriptional regulation, tissue-specific expression, and association with RNA processing networks, MTB may enable context-dependent modulation of the m6A landscape in plants.

Our results support an evolutionary model in which an ancestral MT-A70 gene underwent duplication early in land plant evolution, giving rise to two functionally distinct components of the m6A writer complex. One lineage retained catalytic activity and structural constraint, forming the conserved MTA70 subunit, while the other diverged through loss of catalytic residues and expansion of interaction surfaces, giving rise to the regulatory MTB scaffold [48]. The presence of both components in bryophytes and angiosperms indicates that this functional division was established prior to the diversification of major land plant lineages and has been maintained over hundreds of millions of years.

This model mirrors the organizational logic observed in the mammalian METTL3–METTL14 complex, where a catalytic subunit operates in concert with a structurally related but non-catalytic partner [5,6]. However, the additional regulatory complexity observed in plant MTB proteins, including their diverse promoter architecture and tissue-specific expression patterns, suggests that plants have further adapted this system to integrate developmental and environmental signaling inputs.

The structural and computational analyses presented here provide a framework for experimentally testing this model. The predicted Arabidopsis MTA70–MTB interaction interface, particularly the contributions of residues such as Lys746 and Lys637, offers specific targets for site-directed mutagenesis and biochemical validation. Measuring the effects of these mutations on complex formation and m6A deposition will be critical for confirming the functional relevance of the predicted interface. Structural characterization of the plant writer complex by cryo-electron microscopy or X-ray crystallography remains an important goal and would provide the atomic-resolution data needed to validate the interface geometry predicted here.

Beyond validating the interaction interface, future studies should explore how MTB-mediated regulation influences m6A deposition at specific developmental stages and under environmental stress conditions. The conserved enrichment of MTB expression in reproductive tissues highlights a role in fine-tuning epitranscriptomic regulation during key developmental transitions. Integrating transcriptome-wide m6A profiling with MTB perturbation experiments will help define the regulatory scope of MTB and its contribution to plant gene expression networks.

Several limitations of this study should be noted. All structural models are derived from AlphaFold2 predictions rather than experimental structures; interface residues with pLDDT < 70 in AtMTA70 should be interpreted with caution pending experimental validation. HADDOCK docking results and mCSM-PPI2 alanine scanning predictions require experimental confirmation by co-immunoprecipitation or site-directed mutagenesis studies. FUBAR selection analysis of MTB was restricted to the conserved C-terminal MT-A70-like domain due to high sequence length variation in full-length MTB sequences. Expression data were retrieved from publicly available databases without independent experimental validation, and cross-dataset comparisons are subject to differences in normalization methods. Finally, gymnosperms and ferns could not be included due to incomplete MT-A70 family annotation in available genome resources. Taken together, the structural, evolutionary, and expression analyses presented here support a model in which MTB acts as a regulatory scaffold within the plant m6A writer complex. While the predicted interaction interface remains to be experimentally validated, the concordance observed across independent analyses strengthens confidence in this model. The enrichment of MTB expression in reproductive and stress-related contexts further suggests that its role may extend beyond structural support to include context-dependent regulation of m6A deposition. These findings provide a foundation for future experimental studies aimed at understanding how epitranscriptomic regulation is integrated with plant development and environmental responses.

## 5. Conclusions

This study provides an integrative computational framework for understanding the evolutionary and structural basis of functional divergence within the plant MT-A70 family. Phylogenomic reconstruction across 15 representative lineages, including lycophyte and charophyte outgroups, indicates that the division between the catalytic MTA70 subunit and the non-catalytic MTB scaffold predates the separation of major land plant lineages, identifying this as an ancient and conserved organizational feature of the m6A writer complex. Comparative analyses of gene structure, domain architecture, conserved motifs, and structural models consistently support selective constraint at functionally critical residues in MTA70 and relaxed structural constraints in MTB. FUBAR codon-based selection analysis confirmed pervasive purifying selection in the MTB C-terminal scaffold domain (37 sites, *p* ≥ 0.9; β/α = 0.146) alongside site-specific constraint in MTA70 (2 sites, *p* ≥ 0.9). Protein-protein docking predicted a stable MTA70-MTB heterodimer interface, with Lys746 and Lys637 of MTB identified as primary hotspot residues by computational alanine scanning. Expression profiling and promoter analysis further revealed that MTB is subject to tissue-specific and stress-responsive regulation, particularly in reproductive tissues, suggesting a role in coupling m6A deposition to developmental and environmental cues. These findings establish experimentally testable predictions for future biochemical and structural validation of the plant m6A writer complex. Independent blind docking using HDOCK confirmed convergence on the MTB C-terminal interaction surface, and statistical promoter analysis revealed significant jasmonate-responsive element enrichment in MTB promoters, together providing multi-layered evidence in support of the regulatory specialization of MTB.

## Figures and Tables

**Table 1:** Key interface residues contributing to MTA70–MTB interaction identified by computational alanine scanning. ΔΔG values were predicted using mCSM-PPI2. Negative ΔΔG values indicate destabilization of the protein–protein interaction upon mutation. Residues with ΔΔG ≤ −1.0 kcal/mol are classified as strongly destabilizing. pLDDT values reflect per-residue AlphaFold2 model confidence. Gly116 of MTA70, which forms the central hydrogen bond with Glu628 of MTB, is not included in this table because glycine-to-alanine substitution does not introduce a side chain, precluding meaningful ΔΔG estimation by mCSM-PPI2; its interfacial role is assessed from docking geometry rather than mutational energetics. Asp103 of MTA70 is not included as its predicted ΔΔG fell below the reporting threshold (ΔΔG < 0.5 kcal/mol); additionally, its low pLDDT (29.60) reduces confidence in energy predictions at this position. mCSM-PPI2 provides point estimates of ΔΔG without formal confidence intervals; values represent relative rankings of destabilizing effect and should not be interpreted as precise thermodynamic measurements.

Chain	Residue	Position	Mutation	pLDDT	Distance (Å)	ΔΔG (kcal/mol)	Effect
B	LYS	746	ALA	98.19	3.750	−1.527	Strong destabilizing
B	LYS	637	ALA	97.69	2.687	−1.326	Strong destabilizing
A	PRO	118	ALA	89.56	3.178	−0.928	Destabilizing
B	TRP	704	ALA	98.38	3.663	−0.857	Destabilizing
B	LYS	702	ALA	98.00	2.650	−0.780	Destabilizing
B	LEU	633	ALA	98.94	3.231	−0.753	Destabilizing
B	GLY	747	ALA	96.69	4.224	−0.687	Destabilizing
B	GLU	626	ALA	92.94	2.986	−0.659	Destabilizing
A	ASP	108	ALA	27.60	2.687	−0.601	Destabilizing
B	LYS	673	ALA	96.19	4.707	−0.613	Destabilizing

## Data Availability

The original contributions presented in this study are included in the article/Supplementary Materials. Further inquiries can be directed to the corresponding author.

## Supplementary Materials

The following supporting information can be downloaded at: https://www.mdpi.com/article/10.3390/cimb48070722/s1.
