# Supplementary material for: Evolutionary Divergence, Predicted Interaction Interface, and Regulatory Specialization of MTB as a Non-Catalytic Scaffold in the Plant m6A Writer Complex
Source: Curr Issues Mol Biol. 2026 Jul 15;48(7):722. doi: 10.3390/cimb48070722 (PMC13408939; doi:10.3390/cimb48070722)
Supplement: Supplementary file 1 [file cimb-48-00722-s001.zip › cimb-4366037-supplementary.pdf]

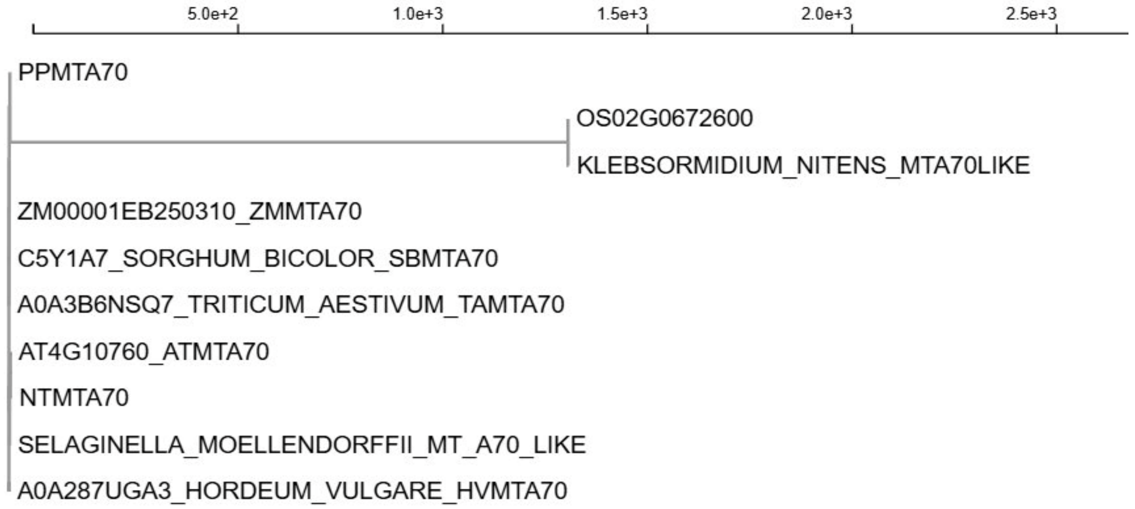

Supplementary Figure S1. Phylogenetic tree inferred by Datamonkey from 12 MTA70 CDS sequences prior to FUBAR analysis. Branch lengths proportional to substitutions per site. KnMTA70 (*Klebsormidium nitens*) and SmMTA70like (*Selaginella moellendorffii*) represent charophyte algal and lycophyte outgroups.

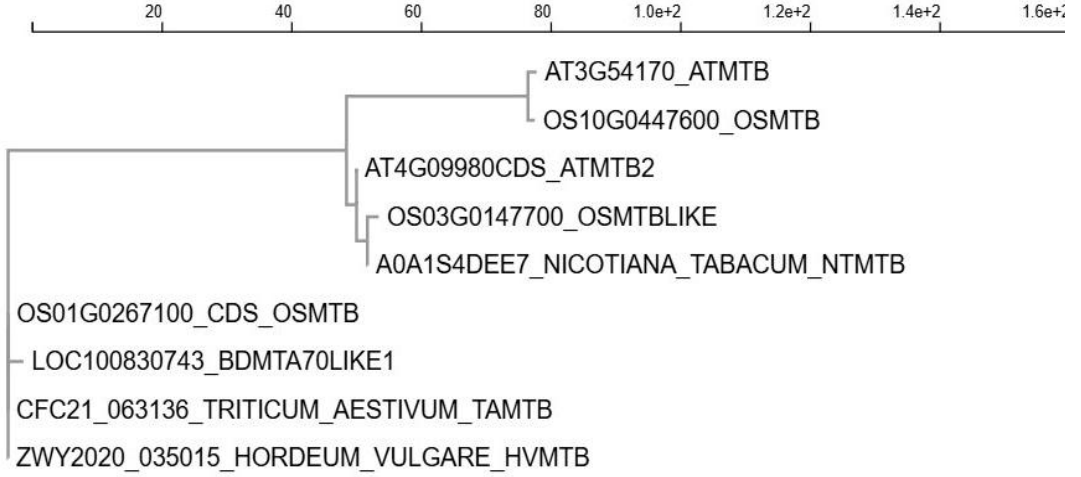

Supplementary Figure S2. Phylogenetic tree inferred by Datamonkey from 9 MTB C-terminal domain sequences prior to FUBAR analysis. Branch lengths proportional to substitutions per site. Sequences represent the conserved C-terminal MT-A70-like scaffold domain (330 aa) of MTB homologs from 9 diverse plant species.

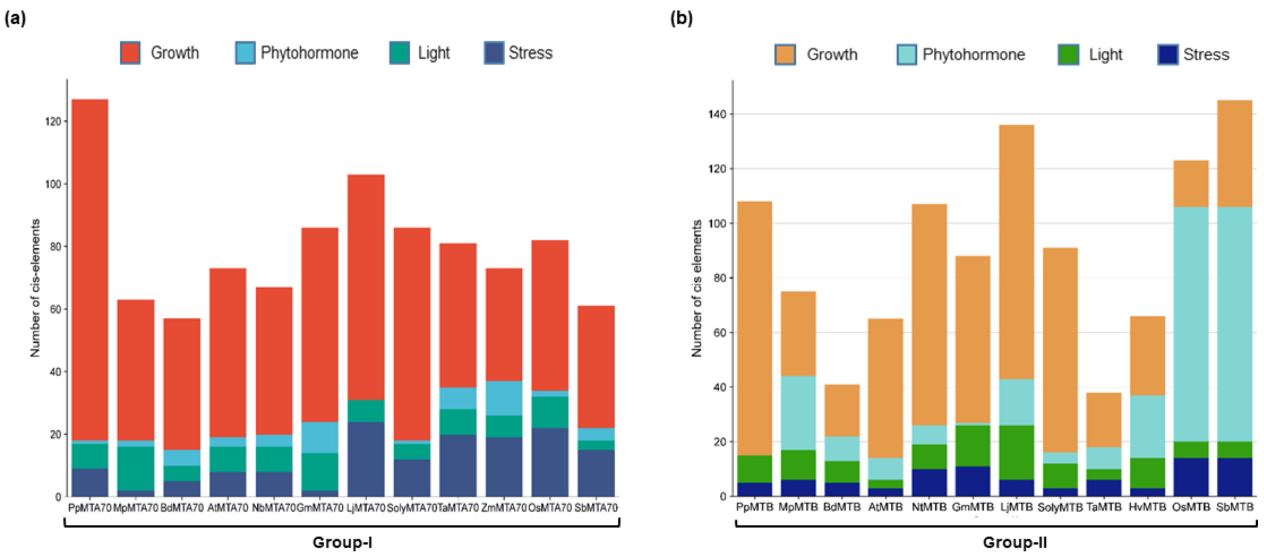

Supplementary Figure S3. *Cis*-regulatory element composition of MTA70 and MTB promoter regions across plant species. *Cis*-acting regulatory elements identified in the promoter regions of MTA70 (a) and MTB (b) genes using the PlantCARE database. Promoter sequences comprising 1,500 bp upstream of the start codon were retrieved from EnsemblPlants, and NCBI genomic resources. Bar graph showing distribution of cis-elements and classified into four functional categories: growth and development, phytohormone response, light responsiveness, and stress response. The proportion and diversity of *Cis*-elements reveal both conserved and lineage-specific regulatory elements regulating transcriptional control of MTA70 and MTB genes. MTA70 genes display relatively conserved, growth-dominated regulatory profiles, whereas MTB genes exhibit greater inter-species variability with enrichment of hormone, light, and stress-responsive elements. Species includes: Pp, *Physcomitrium patens*; Mp, *Marchantia polymorpha*; Bd, *Brachypodium distachyon*; At, *Arabidopsis thaliana*; Nt, *Nicotiana tabacum*; Gm, *Glycine max*; Hv, *Hordeum vulgare*; Lj, *Lotus japonicus*; Soly, *Solanum lycopersicum*; Ta, *Triticum aestivum*; Zm, *Zea mays*; Os, *Oryza sativa*; Sb, *Sorghum bicolor*.

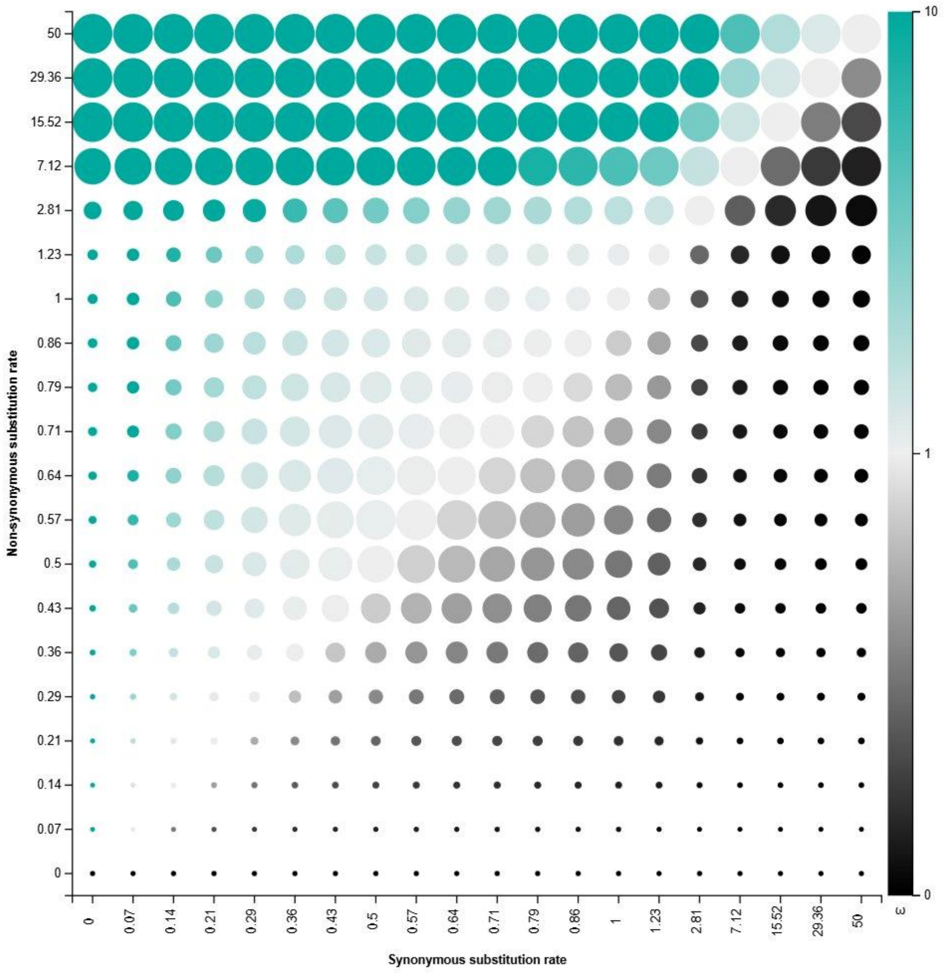

Supplementary Figure S4. FUBAR posterior probability grid for MTA70 coding sequences (810 sites, 12 taxa). Teal circles: purifying selection (alpha > beta). Dark circles: positive selection (alpha < beta). Circle size indicates posterior probability mass. Two sites reached P >= 0.9 for purifying selection (Sites 1 and 20).

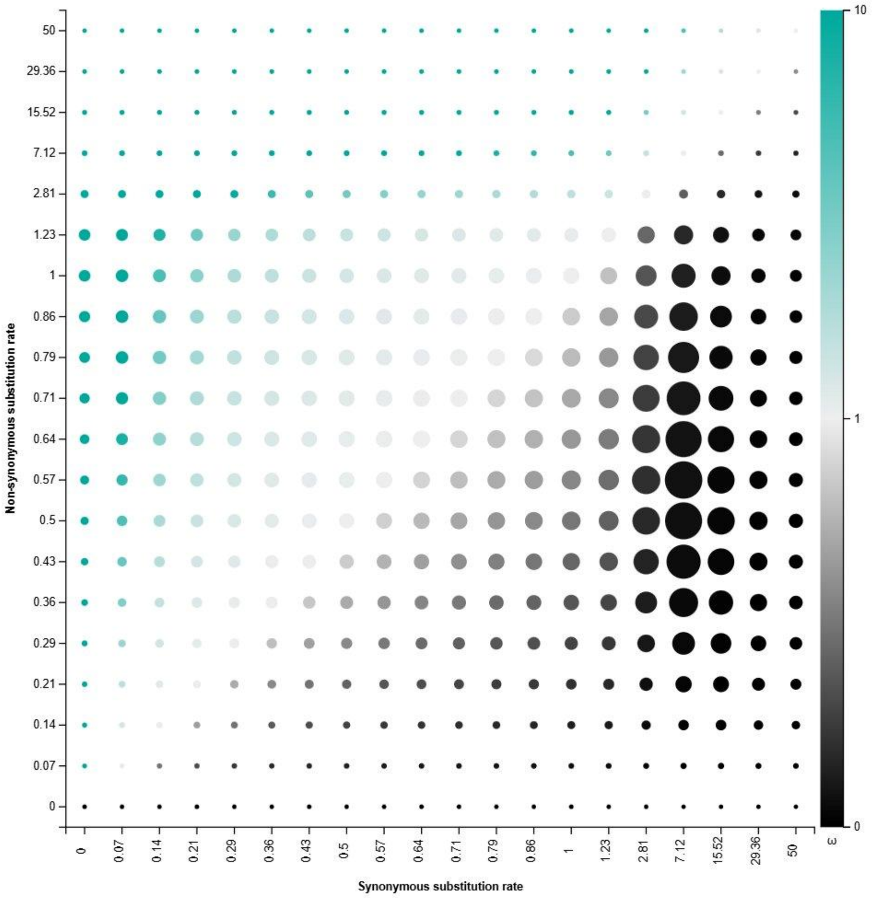

Supplementary Figure S5. FUBAR posterior probability grid for MTB C-terminal MT-A70-like domain (330 sites, 9 taxa). Large dark circles in lower-left quadrant (high alpha, low beta) indicate strong purifying selection across 97% of sites. 37 sites reached P >= 0.9 for purifying selection; no sites under positive selection. Substantially stronger constraint than MTA70 (compare Figure S4), consistent with the essential scaffold function of the MTB C-terminal domain.

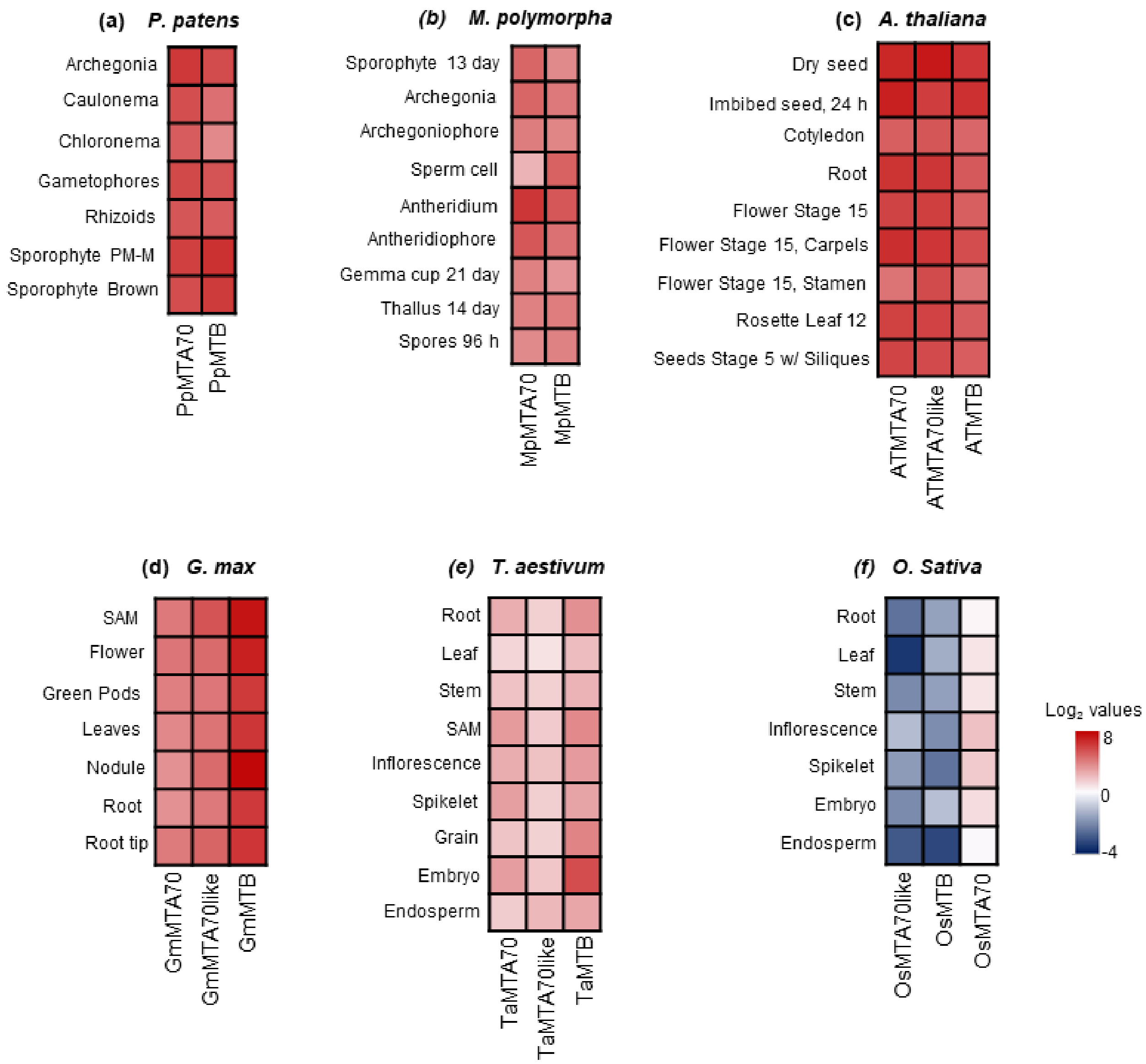

Supplementary Figure S6. Expression patterns of MT-A70 and MTB genes in various plant species. Expression of MTA70 and MTB family genes from various plant species at different stages of development. The TPM and FPKM levels were obtained from public RNA-seq database, and normalized by $\log_2$ transformation for: a) *P. patens, b) M.polymorpha,* c) *A.thaliana*, d). *G.max*, e) *T.aestivum*, and f) *O.sativa.* The expression levels are shown on color scale from blue (low) to red (high) using heatmap visualization tool.

(a)

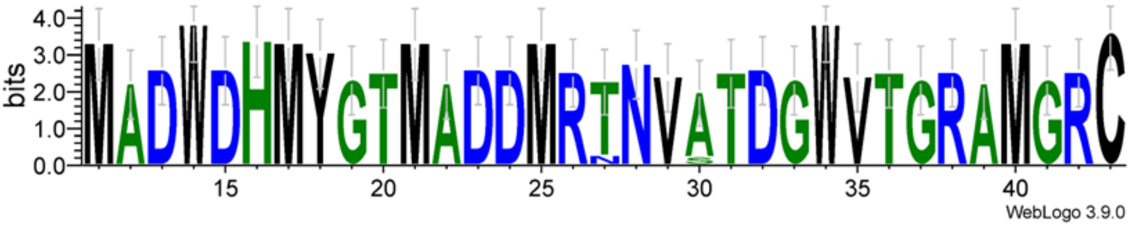

(b)

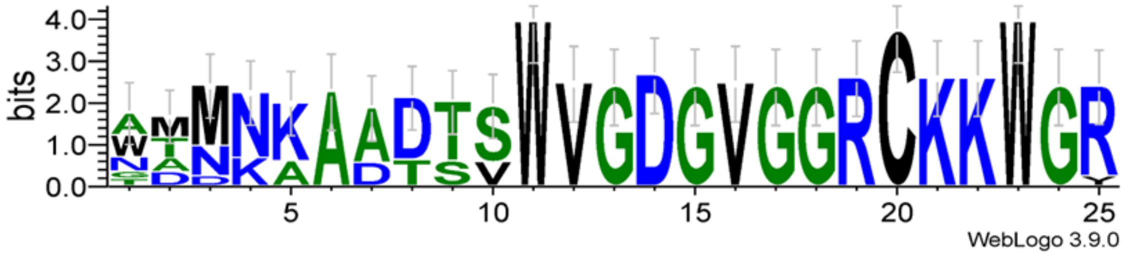

Supplementary Figure S7. Conservation of catalytic domain motifs in plant MT-A70 and regulatory motifs in plant MT-A70-like/MTB proteins. Sequence logos of conserved amino acid residues within the catalytic domain of MT-A70 (a) and MT-A70-like/MTB (b) proteins across representative land plant lineages. The MTA70 logo was created utilizing sequences from *B. distachyon*, *Z. mays*, *O. sativa*, *N. tabacum*, *T. aestivum*, *S. bicolor*, *H. vulgare*, *G. max*, *A. thaliana* (angiosperms), *M. polymorpha* (liverwort), and *P. patens* (moss). The MTA70-like/MTB logo incorporates elements from *O. sativa*, *H. vulgare*, *T. aestivum*, *B. distachyon*, *N. tabacum*, *G. max*, and *A. thaliana*. WebLogo3 generated logos derived from multiple sequence alignments of motifs identified by MEME. The size of the letters indicated the extent to which the residues were maintained (bits). The significant conservation of key residues in the MTA70 catalytic domain among terrestrial plants indicates the maintenance of N^6-adenosine methyltransferase activity. The unique yet conserved motif architecture of MTA70-like/MTB proteins indicates that they serve different functions while retaining the essential characteristics associated with methyltransferases.

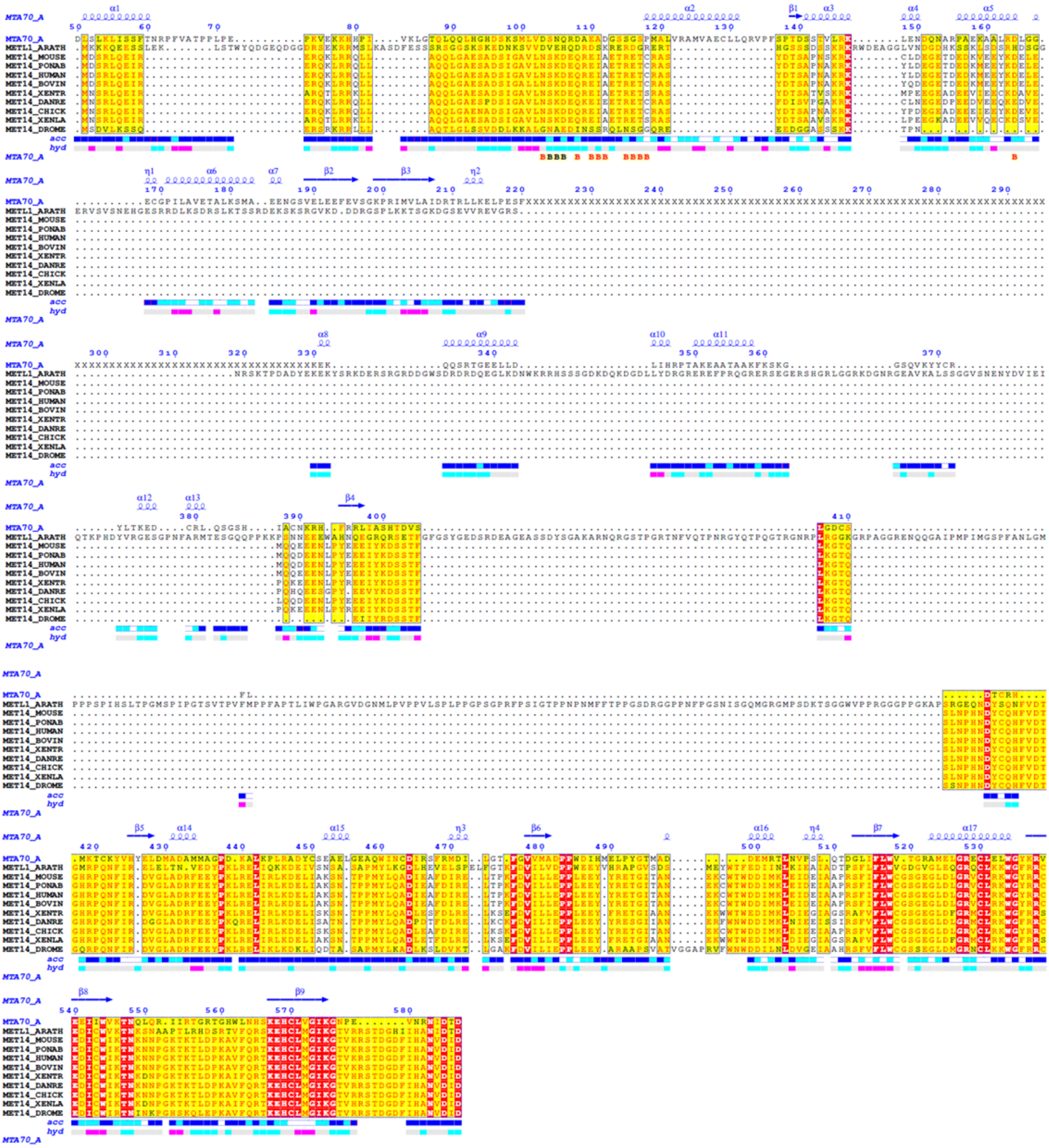

Supplementary Figure S8. ENDscript sequence–structure alignment of the *Arabidopsis thaliana* MTA70–MTB complex against MT-A70 family homologues (MTA70, MTB, and MTA70-like sequences) from representative plant and animal species. Conserved residues are highlighted according to the standard ENDscript colour scheme, with secondary-structure elements shown above the alignment. The alignment illustrates conservation of the C-terminal MT-A70 region across species.

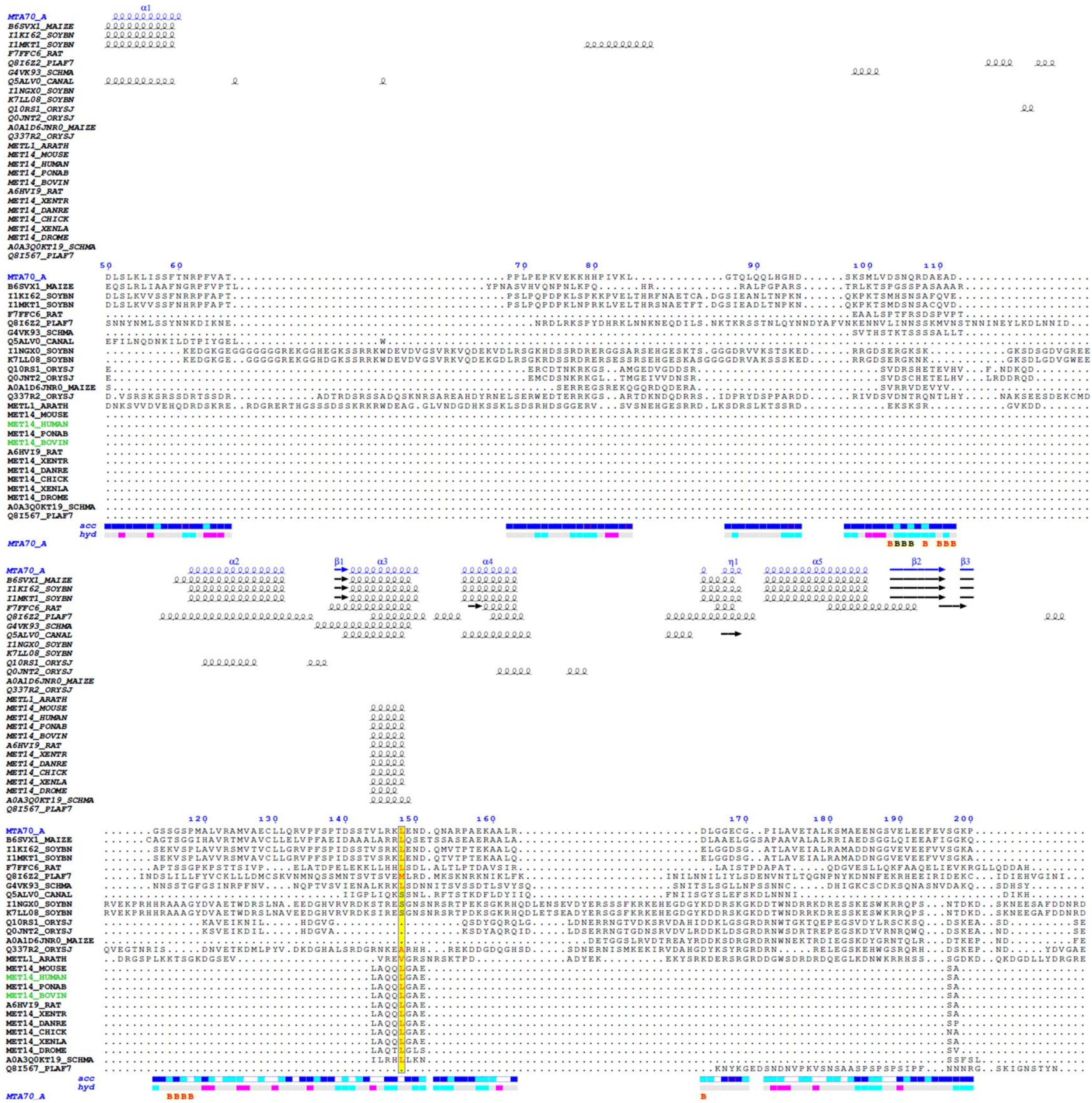

continued to next page…

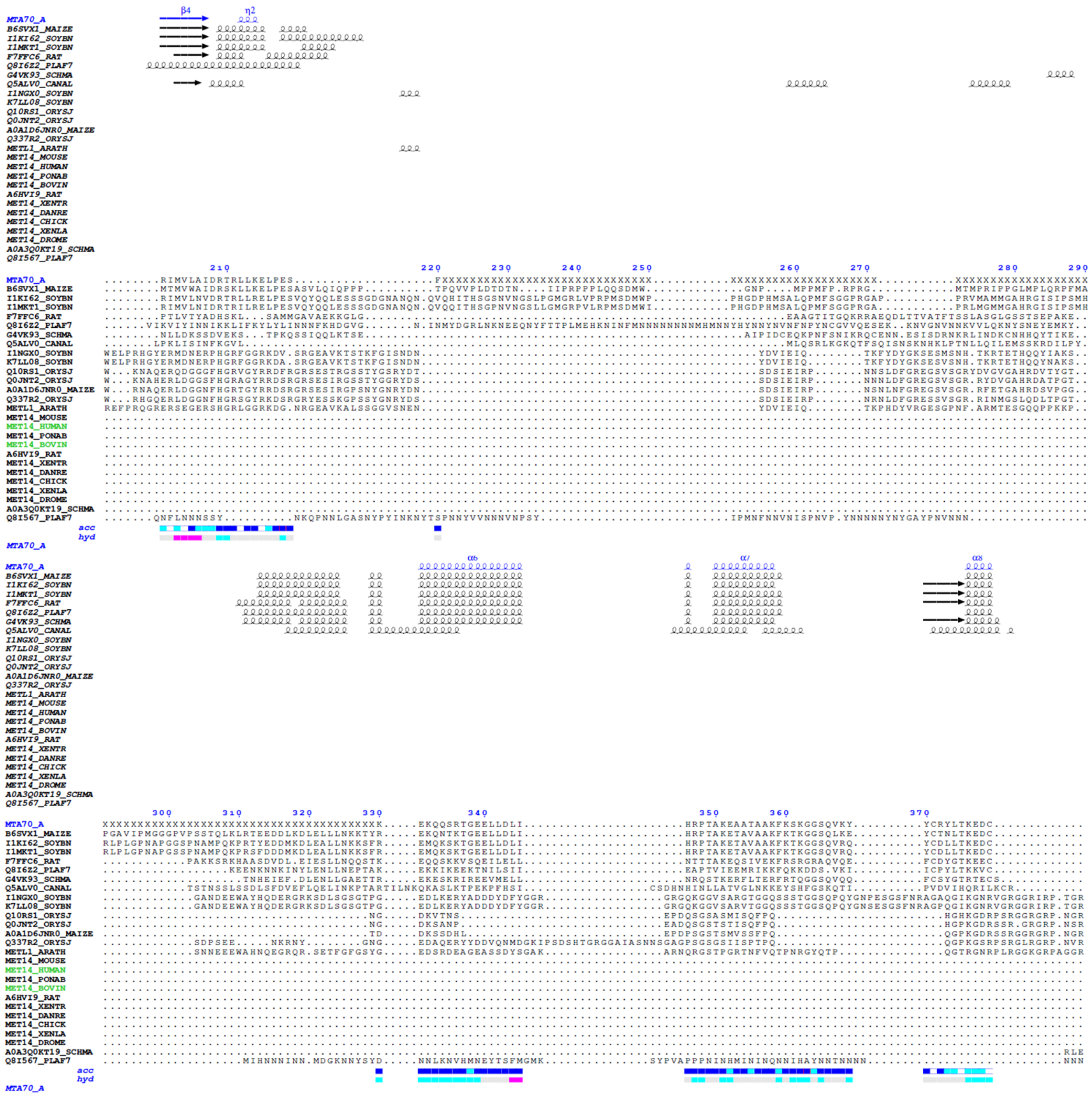

continued to next page…

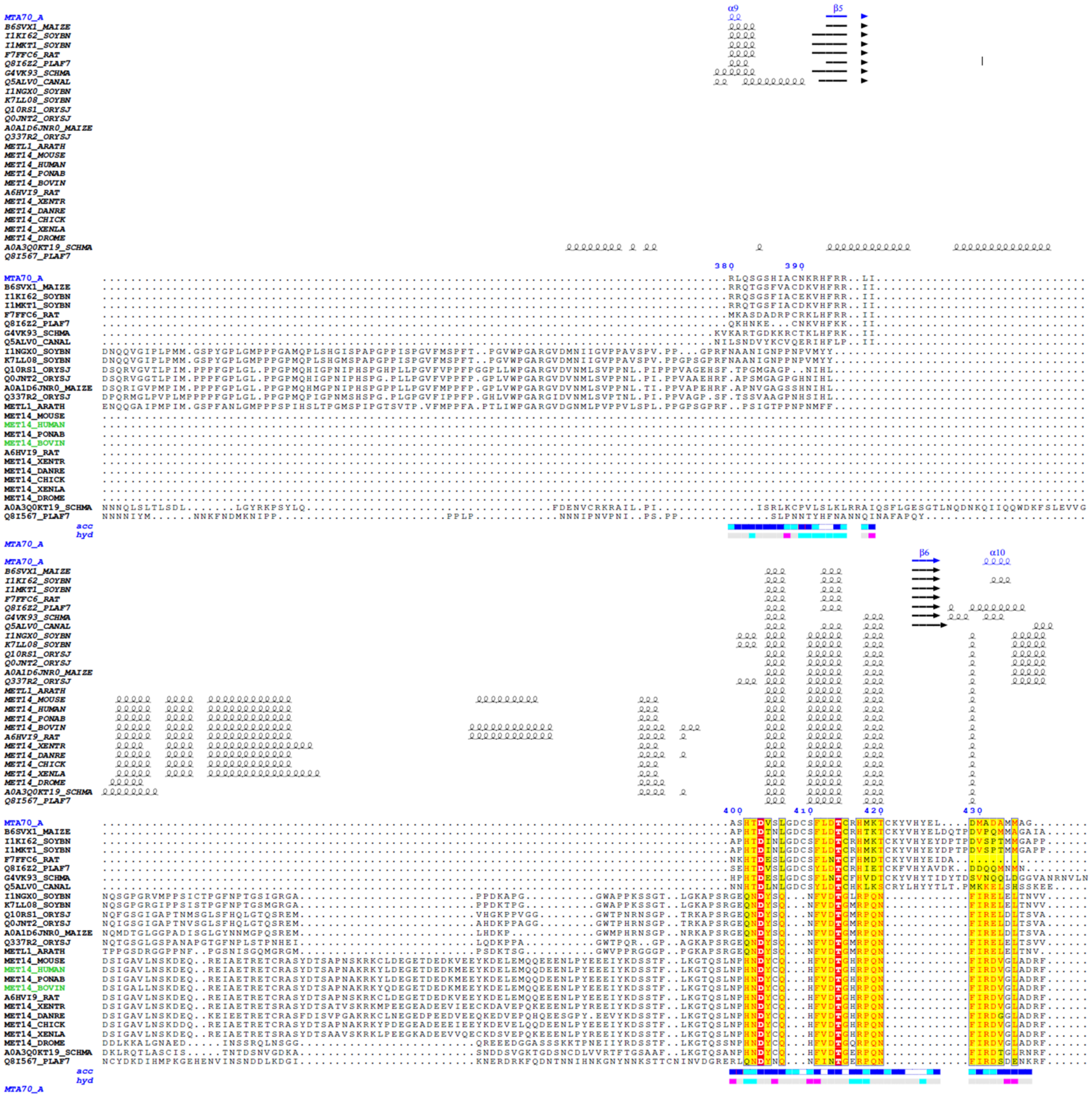

Continued to next page…

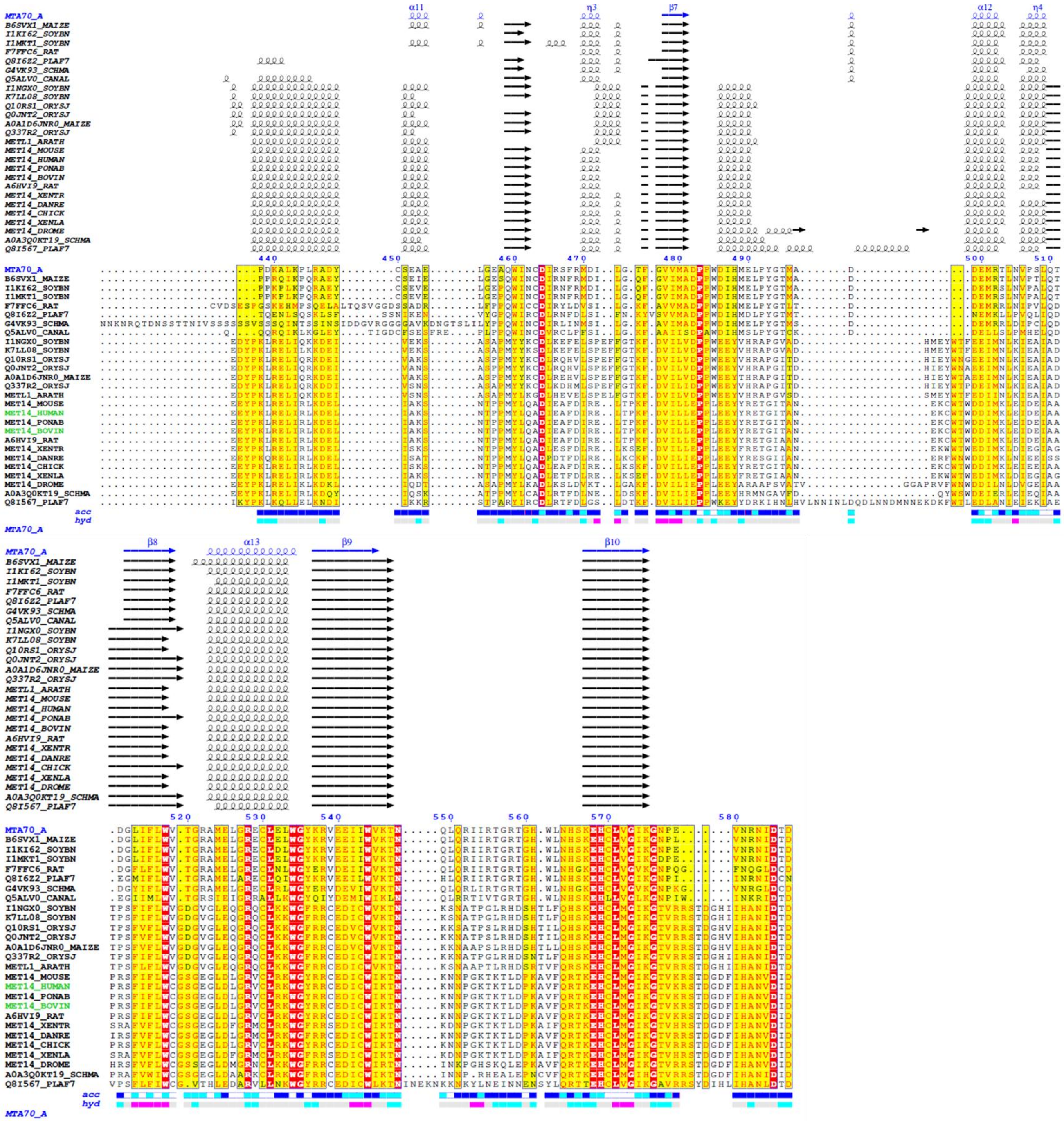

Supplementary Figure S9. ESPript sequence–structure alignment of the *Arabidopsis thaliana* MTA70–MTB complex against an expanded, multi-species panel of MT-A70 family homologues (MTA70, MTB, and MTA70-like sequences) from diverse plant and animal lineages. Conserved residues are highlighted according to the standard ESPript colour scheme, with secondary-structure elements shown above the alignment. The alignment is presented across multiple pages and illustrates conservation of the C-terminal MT-A70 region across species. (full length alignment: page 8-11)

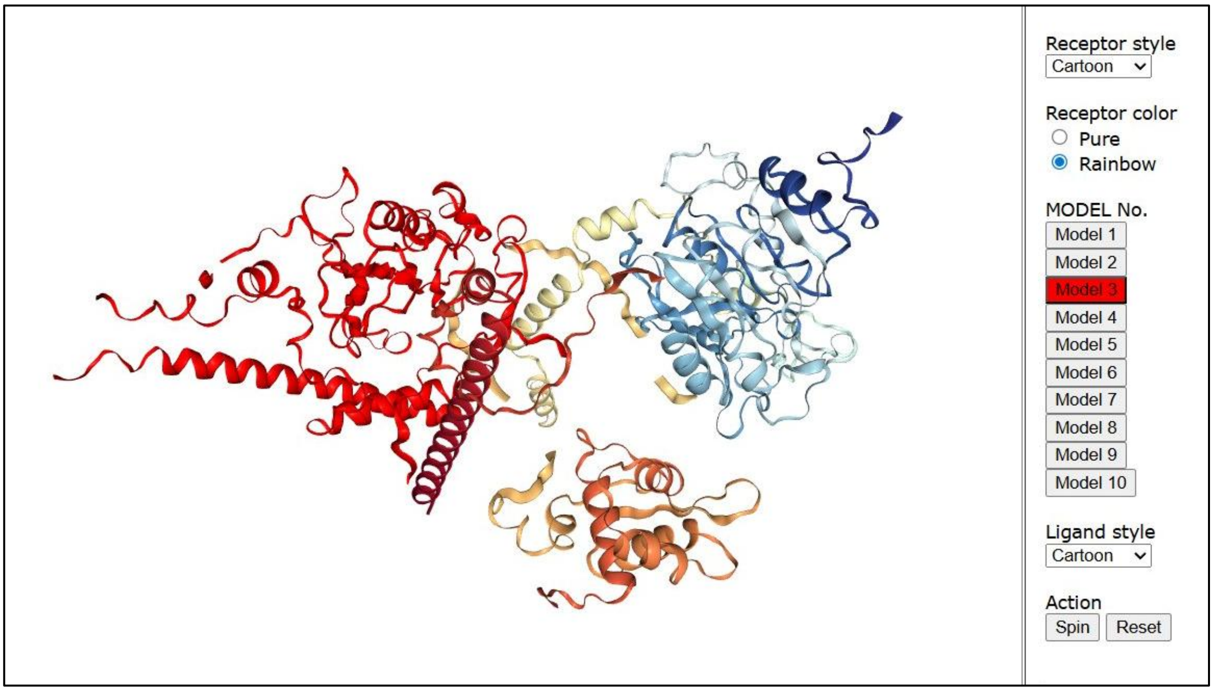

Supplementary Figure S10. HDOCK blind docking prediction of the AtMTA70-AtMTB complex (Model 3; docking score -266.55, confidence 0.9114)**.** Blind docking performed without specifying interface residues. Receptor (AtMTA70) in rainbow; ligand (AtMTB) in blue-white. Both Model 1 (score -327.00, confidence 0.9718) and Model 3 consistently predict the C-terminal domain of MTB (residues 604-774) as the primary interaction surface.

Supplementary Table S1. Domain architecture of MTA70 and MTA70-like/MTB proteins across representative plant lineages identified using Conserved Domain Database (CDD) analysis.

Accession ID	Protein name	Species	Domain type	Domain name	Full length	Accession	From–To (aa)	E-value	Bitscore	Superfamily
A0A2K1LAL9	PpMTA70	Physcomitrium patens	Specific	MT-A70	810	pfam05063	559–719	3.94E-71	230.78	cl01947
A0A2R6X4X1	MpMTA70	Marchantia polymorpha	Specific	MT-A70	816	pfam05063	561–721	6.24E-74	238.49	cl01947
A0A0Q3FME1	BdMTA70	Brachypodium distachyon	Specific	MT-A70	710	pfam05063	494–654	1.80E-73	235.41	cl01947
AT4G10760	AtMTA70	Arabidopsis thaliana	Specific	MT-A70	685	pfam05063	476–636	6.28E-75	238.49	cl01947
A0A1S3ZMH3	NtMTA70	Nicotiana tabacum	Specific	MT-A70	748	pfam05063	503–663	2.27E-74	238.49	cl01947
A0A1S3ZV37	NtMTA70like	Nicotiana tabacum	Specific	MT-A70	749	pfam05063	504–664	2.31E-74	238.49	cl01947
LOC100788519	GmMTA70	Glycine max	Specific	MT-A70	762	pfam05063	516–676	1.27E-74	239.26	cl01947
LOC100782695	GmMTA70like	Glycine max	Specific	MT-A70	761	pfam05063	515–675	7.37E-75	240.03	cl01947
A0A287UGA3	HvMTA70	Hordeum vulgare	Specific	MT-A70	736	pfam05063	520–680	9.96E-74	236.56	cl01947
A0A3B6NSQ7	TaMTA70	Triticum aestivum	Specific	MT-A70	702	pfam05063	486–646	1.40E-73	235.41	cl01947
A0A3B6PPP5	TaMTA70like	Triticum aestivum	Specific	MT-A70	696	pfam05063	480–640	1.03E-73	235.41	cl01947
A0A3B6QIH5	TaMTA70like1	Triticum aestivum	Specific	MT-A70	702	pfam05063	486–646	1.18E-73	235.41	cl01947
B6SVX1	ZmMTA70	Zea mays	Specific	MT-A70	704	pfam05063	488–648	8.26E-75	238.49	cl01947
Q6EU10	OsMTA70	Oryza sativa	Specific	MT-A70	706	pfam05063	491–651	1.56E-74	238.1	cl01947
Q10RS1	OsMTA70like1	Oryza sativa	Superfamily	MT-A70	677	cl01947	421–597	8.66E-57	189.95	cl01947
Q0JNT2	OsMTA70like	Oryza sativa	Superfamily	MT-A70	764	cl01947	529–705	1.26E-56	191.11	cl01947
At4g09980	AtMTB	Arabidopsis thaliana	Superfamily	MT-A70	775	cl01947	638–814	4.75E-56	191.11	cl01947
A0A1S4DEE7	NtMTB	Nicotiana tabacum	Superfamily	MT-A70	1160	cl01947	827–1003	8.96E-56	191.11	cl01947
A0A1U7X345	NtMTBlike	Nicotiana tabacum	Superfamily	MT-A70	1086	cl01947	769–945	1.01E-56	193.8	cl01947

KAL2995502	GmMTB	Glycine max	Superfamily	MT-A70	908	cl01947	575–753	8.34E-58	195.73	cl01947
Os10g0447600	OsMTB1	Oryza sativa	Superfamily	MT-A70	1013	cl01947	725–901	2.61E-59	200.74	cl01947
CFC21_063136	TaMTB	Triticum aestivum	Superfamily	MT-A70	884	cl01947	649–825	8.80E-56	189.95	cl01947
ZWY2020_035015	HvMTB	Hordeum vulgare	Superfamily	MT-A70	764	cl01947	572–724	3.50E-48	167.99	cl01947

Supplementary Table S2. Cis-acting regulatory elements identified in promoter regions of MTA70 and MTA70-like/MTB genes across representative plant species using PlantCARE. The data obtained from this table is used to generate Figure 2b.

**Function**	**Site Name**	PpMTA70	MpMTA70	BdMTA70	AtMTA70	NbMTA70	GmMTA70	LjMTA70	SolyMTA70	TaMTA70	ZmMTA70	OsMTA70	SbMTA70
Growth and development (Group I)	A-box	2		3						1		1	
	CAAT-box	33	33	22	22	28	27	35	14	30	29	28	21
	CAT-box	2		1	2				1				
	CCAAT-box	1		1			1			1	1		1
	GCN4_motif					1							
	NON-box												
	O2-site					1		1					2
	RY-element												
	TATA-box	60	11	14	26	15	28	36	50	14	6	17	13
	AT-rich element	10	1	1	4	1	5		3			2	2
	circadian	1				1	1						
Growth and development Total	668	109	45	42	54	47	62	72	68	46	36	48	39
Function	**Site Name**	PpMTA70	MpMTA70	BdMTA70	AtMTA70	NbMTA70	GmMTA70	LjMTA70	SolyMTA70	TaMTA70	ZmMTA70	OsMTA70	SbMTA70
Phytohormone (Group I)	ABRE			2	1	1	5			3	3		3
	AuxRR-core												1
	CGTCA-motif		2	1	1	1	1			1	3		
	GARE-motif												
	TGA-element												
	P-box				1								

	HD-Zip 3												
	TATC-box	1											
	TCA-element					1	1		1	2		1	
	TGA-box			1			2				2		
	TGACG-motif			1		1	1			1	3	1	
Phytohormone responsive elements Total	50	1	2	5	3	4	10	0	1	7	11	2	4
Function	**Site Name**	PpMTA70	MpMTA70	BdMTA70	AtMTA70	NbMTA70	GmMTA70	LjMTA70	SolyMTA70	TaMTA70	ZmMTA70	OsMTA70	SbMTA70
Light (Group I)	ACE												
	AE-box		1										
	ATCT-motif												1
	Box 4	2	3	1	1	3	3		4		1	2	
	Box II						1						
	Box II -like sequence												
	CAG-motif												
	chs-CMA1a								1	1			
	GA-motif										1		
	GATA-motif		1			1						1	
	GATT-motif												
	3-AF1 binding site		1										
	GT1-motif	1				2		1					
	ATC-motif											1	

	MRE	1	6	1	3		4	6		1		6	
	G-box			2		1	4			5	3		1
	GTGGC-motif		1										
	I-box	1									2		1
	LAMP-element	1			1								
	sbp-CMA1c												
	Sp1		1							1			
	TCCC-motif												
	TCT-motif	2		1	3	1							
Light response elements Total	95	8	14	5	8	8	12	7	5	8	7	10	3
Function	**Site Name**	PpMTA70	MpMTA70	BdMTA70	AtMTA70	NbMTA70	GmMTA70	LjMTA70	SolyMTA70	TaMTA70	ZmMTA70	OsMTA70	SbMTA70
stress (Group I)	ARE	3	1	3	4	1		3	1	3	1		2
	TC-rich	2						1					
	GC-motif			2					1				1
	LTR				2	1							
	MBS	4	1		2	6	2	6	4	1	5	4	7
Stress responsive elements Total	74	9	2	5	8	8	2	24	12	20	19	22	15
Function	**Site Name**	PpMTB	MpMTB	BdMTB	AtMTB	NtMTB	GmMTB	LjMTB	SolyMTB	TaMTB	HvMTB	OsMTB	SbMTB
Growth and development (Group II)	A-box											1	
	CAAT-box	53	16	12	23	12	31	35	42	13	10	5	22
	CAT-box			1			2				1		1
	CCAAT-box		1									1	

	GCN4_motif												
	NON-box					1							
	O2-site		1	1		1			1			3	2
	RY-element												
	TATA-box	39	12	4	27	58	26	25	32	7	17	7	13
	AT-rich element	1	1	1	1	9	2	33			1		1
	circadian												
Growth and development Total	609	93	31	19	51	81	61	93	75	20	29	17	39
Function	**Site Name**	PpMTB	MpMTB	BdMTB	AtMTB	NtMTB	GmMTB	LjMTB	SolyMTB	TaMTB	HvMTB	OsMTB	SbMTB
Phytohormone (Group II)	ABRE		17	6	1			13			10	11	4
	AuxRR-core				1								
	CGTCA-motif		1	1	1	2		1	2	3	5		1
	GARE-motif					1	1					1	
	TGA-element												
	P-box												
	HD-Zip 3												
	TATC-box												
	TCA-element			1	2	1		2		2	2		1
	TGA-box		1		1	1			1		1	2	1
	TGACG-motif		8	1	2	2		1	1	3	5	7	1
Phytohormone responsive elements Total	104	0	27	9	8	7	1	17	4	8	23	86	86

Function	**Site Name**	PpMTB	MpMTB	BdMTB	AtMTB	NtMTB	GmMTB	LjMTB	SolyMTB	TaMTB	HvMTB	OsMTB	SbMTB
	ACE						1						
	AE-box						1	1					
	ATCT-motif					1	1		3				
	Box 4	4				1			1	2			
	Box II							2					
	Box II -like sequence												
	CAG-motif												
	chs-CMA1a				1		1		1				
	GA-motif												
	GATA-motif					1	1					1	
Light (Group II)	GATT-motif												
	3-AF1 binding site												
	GT1-motif	1				3	2						
	ATC-motif	1											
	MRE	3	3	3	1		5	2	1	2	2	2	1
	G-box		7	4				12			9	9	2
	GTGGC-motif												
	I-box					1	2	2	1			1	
	LAMP-element						1	1	1				
	sbp-CMA1c												
	Sp1					2							1

	TCCC-motif		1	1								3	1
	TCT-motif	1			1				1			1	1
Light response elements Total	100	10	11	8	3	9	15	20	9	4	11	6	6
Function	**Site Name**	PpMTB	MpMTB	BdMTB	AtMTB	NtMTB	GmMTB	LjMTB	SolyMTB	TaMTB	HvMTB	OsMTB	SbMTB
stress (Group II)	ARE	2	1	2	2		1	3	2	2		3	1
	TC-rich repeats		1										
	GC-motif												
	LTR					1							
	MBS	3	4	3	1	9	10	3	1	4	3	1	1
Stress responsive elements Total	58	5	6	5	3	10	11	6	3	6	3	14	14

Supplementary Table S3. Expression data (log_2-transformed TPM/FPKM values) of MTA70 and MTB genes across multiple plant species used for heatmap visualization in Supplementary Figure S6.

Tissue	AT4G10760-ATMTA70	AT3G54170-AtMTA70like	AT4G09980 - AtMTB
Dry seed	6.79363582	7.242316682	6.333960351
Imbibed seed, 24 h	7.025250322	6.068455852	6.489286023
Cotyledon	5.000901403	5.29351763	4.76394266
Root	6.411934625	6.297374861	5.204375511
Flower Stage 15	5.882153917	6.010556078	4.996840648
Flower Stage 15, Carpels	6.544887178	6.333244817	5.56924803
Flower Stage 15, Stamen	4.343407822	5.64385619	4.430954271
Rosette Leaf 12	5.964167561	5.91336816	5.124328135
Seeds Stage 5 w/ Siliques	5.854993017	5.661635602	5.065227623
**Tissue**	**Glyma.07g067100 MT-A70**	**Glyma.16g033100 MT-A70-like**	**Glyma.20g161800 MTB**
SAM	4.168321116	5.34091827	7.396176618
Flower	4.290571851	4.621172753	6.99095486
Green_Pods	3.974529312	4.257764969	6.218781168
Leaves	3.675815931	4.339137385	6.313790025
Nodule	3.411426246	4.612942196	7.779194046
Root	3.399171094	4.185866545	6.164504726
Root_tip	4.138323004	4.813524689	6.333781501

**Organs**	**Pp3c1_32590V3.1 (ppMTA70)**	**Pp3c5_5900V1.1 (ppMTB)**	
Archegonia	6.222263604	5.597531174	
Caulonema	5.530133184	4.480265122	
Chloronema	5.10349764	3.656496371	
Gametophores	5.704318678	5.336640446	
Rhizoids	5.300489844	5.091699834	
Sporophyte PM-M	5.979110755	6.459923363	
Sporophyte Brown	5.571373436	6.157447996	
Organs	Mp1g08870 (MTA70)	Mp1g04450 (MTB)	
Sporophyte 13 day	4.781839745	3.622307156	
Archegonia	4.779448582	4.170667237	
Archegoniophore	4.055576151	3.765126562	
Sperm cell	2.340015755	4.927783808	
Antheridium	6.300311913	5.241098248	
Antheridiophore	5.240046414	4.433855003	
Gemma cup 21 day	3.906468081	3.305558514	
Thallus 14 day	3.911910829	4.068009639	
Spores 96 h	3.632322083	3.876743962	
**Tissue**	**TraesCS6A02G254900.1**	**TraesCS6B02G270600.1**	**TraesCS4D02G311200**
Root	2.477677328	1.416839742	3.432959407
Leaf	1.257010618	0.887525271	2.032100843
Stem	1.835924074	1.422233001	2.319039816

SAM	3.139142019	1.604071324	3.657640005
Inflorescence / Spike	2.541019153	1.89917563	3.085764554
spikelets	2.981852653	1.480265122	2.817623258
Grain	1.794935663	1.384049807	3.825785627
Embryo	3.03562391	1.722466024	5.568944145
Endosperm	1.526068812	2.169925001	2.739848103
Tissue	Os03g0147700	Os10g0447600	Os01g0267100
Root (vegetative)	-2.501	-1.635	0.2273
Leaf (Leaf blade vegetative)	-3.57	-1.428	0.7725
Stem (vegetative)	-2.071	-1.657	0.7765
Inflorescence (0.6–1.0 mm)	-1.15	-2.009	1.881
Spikelets (Lemma + Palea 1.5–2.0 mm)	-1.752	-2.4805	1.596
Embryo 7 DAF	-2.05	-1.106	1.028
Endosperm 14 DAF	-2.96	-3.266	0.1382

Supplementary Table S4. ABA-responsive expression of MTA70 and MTB genes across representative plant species derived from publicly available transcriptomic datasets

Species	Gene ID	Gene Name	Tissue	Log2 Fold Change (ABA)	Data Source
Physcomitrium patens	Pp3c5_5900V3.1	MTB	Whole plant	−0.48	Perroud et al., 2018
Physcomitrium patens	Pp3c1_32590V3.1	MTA70	Whole plant	−0.08	Perroud et al., 2018
Marchantia polymorpha	Mp1g04450	MTB	Whole plant	−0.08	Jahan et al., 2018
Marchantia polymorpha	Mp1g08870	MTA70	Whole plant	0.15	Jahan et al., 2018
Oryza sativa	Os10g0447600	MTB	Shoot	0.55	RiceXPro
Oryza sativa	Os03g0147700	MTA70	Shoot	1.3	RiceXPro
Arabidopsis thaliana	AT4G10760	MTA70	Seedlings	−5.58	GEO: GSE127910
Arabidopsis thaliana	AT4G09980	MTB	Seedlings	−5.91	GEO: GSE127910
Hordeum vulgare	HORVU.MOREX.r3.4HG0408030; HvMTB	MTB	Root	−0.31	BarleyExpDB
Hordeum vulgare	HORVU.MOREX.r3.6HG0603200(HvMTA70)	MTA70	Root	−0.38	BarleyExpDB

Supplementary Table S5. FUBAR (Fast Unconstrained Bayesian AppRoximation) selection analysis of MTA70 and MTB coding sequences.

Clade	Sequences (taxa)	Sites Analyzed	Purifying Sites (P≥0.9)	Positive Sites (P≥0.9)	Top Significant Sites	Mean beta/alpha
MTA70 (full CDS)	12	810	2 (P≥0.9); 1 (P≥0.95)	2 (P≥0.9); 0 (P≥0.95)	Site 1: P=0.950, alpha=5.631, beta=0.000 Site 20: P=0.914, alpha=4.173, beta=0.244	2.393 (mean beta > alpha)
MTB (C-terminal MT-A70 domain, 330 aa)	9	330	37 (P≥0.9); 11 (P≥0.95)	0	Site 323: P=0.989, alpha=24.421, beta=0.706 Site 64: P=0.983, alpha=24.528, beta=0.483 Site 91: P=0.966, alpha=22.966, beta=0.653 (+34 additional sites at P>=0.9)	0.146 (97.0% sites beta < alpha)

MTA70 analysis: 12 CDS sequences (full length, 810 codon sites), Datamonkey FUBAR, universal genetic code. MTB analysis: 9 sequences, C-terminal MT-A70-like domain only (330 aa = 990 nt), corresponding to the conserved scaffold domain represented by AT4G09980 (MTB). Purifying selection: P(alpha>beta) >= 0.9; Positive selection: P(alpha<beta) >= 0.9. The MTB domain shows substantially stronger purifying selection (37 sites, beta/alpha = 0.146) compared to MTA70 (2 sites, beta/alpha = 2.393), consistent with strong functional constraint on the non-catalytic scaffold domain.

Supplementary Table S6. Comparison of guided (HADDOCK) and blind (HDOCK) docking predictions for the AtMTA70-AtMTB complex.

Method	Docking Score	Confidence/Z-score	Receptor (MTA70) Interface	Ligand (MTB) Interface Region	Key Shared Residues with HADDOCK	Nearest HADDOCK residue (A)
HADDOCK (guided)	−73.7 ± 3.1 kcal/mol	Z-score: −1.8	Asp108, Pro118 (MT-A70 domain)	Glu626, Leu633, Lys637, Lys673, Lys702, Trp704, Lys746, Gly747	— (reference method)	—
HDOCK Model 1 (blind)	−327.00	0.9718	Res. 50–70, 236–244, 330–344	Res. 612–757 (C-terminal domain)	Leu633 (0.0 A)	Glu626→Val627: 3.8 A; Lys637→Phe638: 3.8 A
HDOCK Model 3 (blind)	−266.55	0.9114	Res. 6–70, 239–240, 341–344	Res. 604–774 (C-terminal domain)	Leu633 (4.0 A), Glu632 (3.1 A)	Glu626→Val627: 2.6 A; Lys637→Phe638: 1.3 A

HADDOCK was guided by conserved MEME motif residues. HDOCK blind docking used full-length AlphaFold2 models without residue specification. Both methods identify the C-terminal domain of MTB (residues 604-774) as the primary interaction surface, with Leu633 predicted by all three methods.

Supplementary Table S7. Statistical comparison of cis-acting regulatory element frequencies between MTA70 and MTB promoters (Mann-Whitney U test).

Cis-element	Biological Function	MTA70 Total (n=12)	MTB Total (n=12)	MTA70 Mean	MTB Mean	Ratio (MTB/MTA70)	p-value (Mann-Whitney U)	Significance
TATA-box	Core promoter	290	267	24.17	22.25	0.92	0.7288	ns
CAAT-box	Core promoter	322	274	26.83	22.83	0.85	0.2718	ns
ABRE	ABA-responsive	18	62	1.5	5.17	3.44	0.3074	ns
TGACG-motif	MeJA/JA-responsive	8	31	0.67	2.58	3.88	0.0252	*
CGTCA-motif	MeJA/JA-responsive	10	17	0.83	1.42	1.7	0.298	ns
JA elements (combined)	MeJA/JA-responsive	18	48	1.5	4	2.67	0.0261	*
G-box	Light-responsive	16	43	1.33	3.58	2.69	0.4401	ns
Box 4	Light-responsive	20	8	1.67	0.67	0.4	0.0504	ns
MRE	Light-responsive	28	25	2.33	2.08	0.89	0.7466	ns
MBS	Drought/stress-responsive	42	43	3.5	3.58	1.02	0.7033	ns
ARE	Anaerobic/stress-responsive	22	19	1.83	1.58	0.86	0.6769	ns
AT-rich element	Developmental	29	50	2.42	4.17	1.72	0.5496	ns

Two-sided Mann-Whitney U tests applied to element counts across 12 species per group. Promoter: 1,500 bp upstream of ATG; PlantCARE score >= 8. JA elements (combined) = CGTCA-motif + TGACG-motif per species. * $p < 0.05$; ns, not significant.

Supplementary Table S8. Quantitative structural superposition of Arabidopsis MTA70 and MTB onto human METTL3 and METTL14 using ChimeraX Matchmaker.

Plant Protein	Human Homolog	AlphaFold Model	Pruned RMSD (A)	Atom Pairs	Alignment Score
AtMTA70 (AF-O82486-F1)	METTL3 (catalytic subunit)	AF-Q86U44-F1	0.333	121	694.7
AtMTB (AF-Q94A14-F1)	METTL14 (non-catalytic subunit)	AF-Q9HCE5-F1	0.561	182	716.8

Superposition performed using Needleman-Wunsch alignment with BLOSUM-62 matrix. Pruned RMSD calculated after iterative exclusion of outlier Ca atom pairs. Full-length AlphaFold2 models used.
